# Quantification of insecticides in commercial seafood sold in East Asian markets: risk assessment for consumers

**DOI:** 10.1007/s11356-022-24413-7

**Published:** 2022-12-14

**Authors:** Lucia Ivorra, Patricia G. Cardoso, Shek Kiu Chan, Catarina Cruzeiro, Karen Tagulao

**Affiliations:** 1 Institute of Science and Environment, ISE—University of Saint Joseph, Macao, SAR China; 2 CIIMAR/CIMAR—Interdisciplinary Centre for Marine and Environmental Research, University of Porto, Matosinhos, Portugal; 3 Helmholtz Zentrum München, German Research Centre for Environmental Health, GmbH, Research Unit Comparative Microbiome Analysis, Ingolstaedter Landstrasse 1, 85764 Neuherberg, Germany

**Keywords:** Bivalves, Crustaceans, Health risk assessment, Mudskippers, Organochlorine pesticides

## Abstract

The extraction of 21 insecticides and 5 metabolites was performed using an optimized and validated QuEChERS protocol that was further used for the quantification (GC–MS/MS) in several seafood matrices (crustaceans, bivalves, and fish-mudskippers). Seven species, acquired from Hong Kong and Macao wet markets (a region so far poorly monitored), were selected based on their commercial importance in the Indo-Pacific region, market abundance, and affordable price. Among them, mussels from Hong Kong, together with mudskippers from Macao, presented the highest insecticide concentrations (median values of 30.33 and 23.90 ng/g WW, respectively). Residual levels of fenobucarb, DDTs, HCHs, and heptachlors were above the established threshold (10 ng/g WW) for human consumption according to the European and Chinese legislations: for example, in fish-mudskippers, DDTs, fenobucarb, and heptachlors (5-, 20- and tenfold, respectively), and in bivalves, HCHs (fourfold) had higher levels than the threshold. Risk assessment revealed potential human health effects (e.g., neurotoxicity), especially through fish and bivalve consumption (non-carcinogenic risk; ΣHQ_LT_ > 1), and a potential concern of lifetime cancer risk development through the consumption of fish, bivalves, and crustaceans collected from these markets (carcinogenic risk; ΣTCR > 10^–4^). Since these results indicate polluted regions, where the seafood is collected/produced, a strict monitoring framework should be implemented in those areas to improve food quality and safety of seafood products.

**Graphical Abstract:**

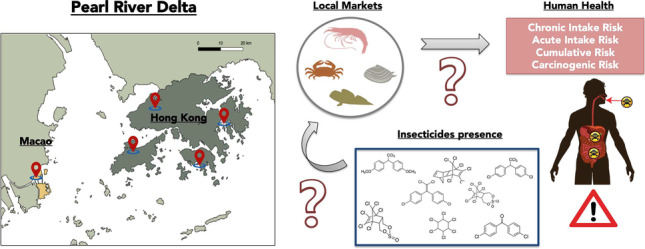

**Supplementary Information:**

The online version contains supplementary material available at 10.1007/s11356-022-24413-7.

## Introduction

The last three decades has witnessed explosive economic growth around the Pearl River Delta (PRD), the southern Chinese region, resulting in environmental deterioration due to rapid industrialization, urbanization, and a massive increase in agricultural lands (Guo et al. 2007; Ouyang et al. 2019). As a result of this agricultural development, there has been a massive increase in pesticide application in this region (Zhang et al. 2002; Yi et al. 2019). Among these pesticides, the organochlorines (OCPs), listed by the United Nations Environmental Programme (2016) as persistent organic pollutants (POPs), are referred to as ubiquitous environmental pollutants due to their extensive applications in the past and their chemical properties (Olisah et al. 2019). Despite the ban of OCPs’ usage (like technical dichlorodiphenyltrichloroethane (DDTs) and hexachlorocyclohexane (HCHs)) in agriculture in the 1980s (Grung et al. 2015), lindane (γ-HCH) and dicofol (synthesized by technical DDTs) were still used for a longer period (Li et al. 2021a, b). Moreover, antifouling paints used in ships have been identified as a potential source of DDTs (as a subproduct of dicofol) (Olisah et al. 2019). According to Liu et al. (2015), China was the largest consumer of dicofol, with a cumulative usage of 19.5 kt (69.1% of the worldwide total) between 2000 and 2012, although its annual domestic usage was reduced by approximately 75% from 2.0 kt in 2000 to 0.53 kt in 2012.

Recent studies show cause for concern regarding the high OCP concentrations in the environment. For example, Tang et al. (2020) found high DDT concentrations (average of 4.03 ng/g) in sediments of a particular region of the Pearl River Estuary (i.e., Humen outlet), representing a medium ecological risk. Moreover, a quite recent publication from Yao et al. (2022) detected high concentrations of total OCPs (average of 65.5 ng/g) in soils of the PRD. Among them, DDTs accounted for the largest share with an average concentration of 34.2 ng/g followed by HCHs (4.52 ng/g). Tang et al. (2018) also revealed a moderate pollution by OCPs in Xijiang River (PRD), with total DDT levels in water and sediments ranging from 0.82 to 2.7 ng/L and 7.1 and 43 ng/g, respectively. Additionally, total HCHs in water and sediment ranged from 3.0 to 53 ng/L and 4.0 and 14 ng/g, respectively. Regarding the air quality in PRD, a recent study from Tian et al. (2021) revealed that DDTs decreased considerably, with a maximum 30-fold reduction from ∼2000 to ∼70 pg/m^3^ since the 2000s. HCB concentrations also declined in the PRD in the last decade from ∼150 to 80 pg/m^3^. However, few studies document pesticide levels on aquatic organisms in this region. In 2003, Wei et al. (2006) quantified several insecticides (HCHs, DDTs, heptachlor, aldrin, endosulfan) in seafood products (i.e., mussels, oysters and shrimps) sampled from the PRD, with results above the threshold (10 ng/g WW) established for human consumption by European (SANCO, 2010) and Chinese legislation (GB 2763-2014 2014). Guo et al. (2008a, b) reported high levels (ranging from 1.3 to 7860 ng/g) of OCPs in more than 70% of the sampled fish (390 individuals) around the Guangdong province. Moreover, Grung et al. (2015), which did an extensive description of OCPs in fish, bivalves, and crustaceans from different Chinese regions, highlighted that the DDT concentrations quantified in fish, from the PRD estuary, are likely to possess ecotoxicological effects. The same was recently documented in a similar study covering 15 provinces and municipalities in China (Chen et al. 2022). These studies already show some concerning levels in the seafood products that might compromise the food resources quality, like fisheries and aquaculture in the surrounding areas.

In addition, the carbamate insecticide fenobucarb was also included in the study, due to its high current use in China (Lan et al. 2019). A recent report indicated a production of fenobucarb of around 4500 tons/year, with 19% production usage, by the largest carbamate production company in China (Yang et al. 2020). This compound has been quantified in water samples from different Chinese regions (Zheng et al. 2016; Xu et al. 2019, 2020), although information regarding its concentrations in seafood samples, as far as we know, is not available.

Macau and Hong Kong, both situated in the PRD Bay, are two densely populated regions (over 22,000 and 6700 hab./km^2^, respectively) that depend on the Hong Kong and Guangdong province fish industry (Chen et al. 2008; AFCD 2021). For example, in Hong Kong, fresh food products are usually purchased from wet markets (Sapugahawatte et al. 2022). It is also known that Hong Kong’s demand and consumption of seafood is substantial, with a per capita average consumption of 66.5 kg, ranking second in Asia in 2017, showing the importance of these markets in the region (World Wildlife Fund n.d.).

These compounds and their subproducts (degradation and metabolite products) are very persistent, and due to their chemical properties (e.g., octanol–water partition coefficient (log K_ow_ > 3), half-life > 60 days), they are prone to bioaccumulate and biomagnify through tropic levels, indirectly affecting top predators (Nakata et al. 2002; Carvalho 2017). It is also known that lipid tissues will influence the accumulation of these insecticides (Sun et al. 2015). Therefore, it is expected that organisms with higher lipid content will present higher concentrations of pesticides (H1).

Due to the high consumption rate of bivalves in China (ca. 14 000 10^3^ tons/year; FAO 2018), and the fact that the complete soft body of these organisms is ingested, it is expected to have higher health risk linked with the ingestion of these animals than other groups (i.e., fish or crustaceans) (H2). Also, their sessile lifestyle and their feeding behaviour—the higher capacity to filter contaminants from the environment dissolved and particulate matter—can potentiate the accumulation of organic pollutants (O´Connor 2002; Monirith et al. 2003; Suárez et al. 2012).

For the first time, this work provides a method for fenobucarb and some relevant OCPs’ metabolite quantification, in seafood. Moreover, due to the economic and cultural importance of seafood in Macao and Hong Kong region, we acquired seven commonly consumed species, from local markets, to assess their quality for human consumption through human health risk assessment. To accomplish this task, we (1) optimized the extraction protocol for several matrices (i.e., fish-mudskippers, bivalves, and crustaceans) acquired from different markets located in the Pearl River Delta and (2) validated the quantification of 21 target insecticides (mainly OCPs).

## Materials and methods

### Seafood acquisition

Seven adult-size species, commonly consumed in South East Asia and Guangdong province (China, SAR), were selected for this work: three species of bivalves (*Meretrix meretrix*, *Anadara subcrenata*, and *Perna viridis)*, three species of crustaceans (*Portunus pelagicus, Scylla serrata*, and *Metapenaeus ensis),* and one species of fish *(Boleophthalmus pectinirostris*). They were acquired from seven local seafood markets in Hong Kong (*N* = 4) and Macao (*N* = 3), in June–August 2018. Market locations and specific information about the purchased seafood are included in the supplementary material (Fig. S1 and Table S1, respectively). In each market, animals were bought from two different suppliers, and two independent samples were analysed per supplier. However, the variety and quantity of seafood samples were slightly different according to the demand of each location. After the acquisition, bivalves and crustaceans were kept in iceboxes, and once in the lab, they were stored at − 80 °C until further analysis. For the fish (mudskippers), the animals were anaesthetized for 1 h (tricaine methanesulfonate (300 mg/L) at 4 °C) and then euthanized by cervical cut, immediately after arriving at the laboratory. This procedure was performed by a certified researcher in practical animal experimentation, aquatic animals (category B), and carried out in accordance with the EU Directive 2010/63/EU, Annex IV, on the protection of animals used for scientific purposes.

### Extraction of 21 insecticides from seafood products

The dispersive extraction method (QuEChERS technique), adapted from Cruzeiro et al. (2016), was used for all seafood matrices. After defrosting, samples were cut and grounded with a high-speed disintegrator (ref. FW80, Faithful, China), and 5 g was then placed into a 50-mL Teflon centrifuge tube (Nalgene Oak Ridge High-Speed, Thermo- Fisher, NY, USA). A fortified sample was prepared by spiking 50 µL of the IS-mix (500 μg/L as a stock solution) and/or 50 µL of each calibration curve solution (16–512 μg/L, as a stock solution) in the homogenate. Samples were let to settle for 5 min before vortex, then 5 mL of CH_3_CN was added and vortexed again. The next steps included vortex and centrifugation between steps 1 and 3 (4 °C, 4024 rcf, 5 min): (1) addition of 2 g MgSO_4_ and 500 mg C_2_H_3_NaO_2_; (2) collection of the upper layer (2.5 mL); (3) addition of 125 mg PSA and 375 mg MgSO_4_; and (4) collection of the final extract (2 mL) and injected within the next 24 h.

All pesticide standards and reagents used in the extraction procedure can be found in the supplementary material file.

### Lipid content

Evaluation of the lipid content (g/g WW and %) was done using the Bligh & Dyer lipid determination method (Smedes and Thomasen 1996): (1) 5 mL of CHCl_3_ and 10 mL of CH_3_OH were added into 5 g of homogenized sample and vortexed during 2 min; (2) 5 mL of CHCl_3_ was added and vortexed again during 30 s, followed by 5 mL of H_2_O and vortexed for additional 30 s; (3) the homogenate was then filtered (0.7-μm glass microfiber filter, Whatman, Germany) and the filtrate collected into a graduated cylinder; and (4) after 5 min, the filtrate was settled and separated into two layers, with CHCl_3_ as the lower phase.

The lipid content was calculated by dividing the weight of five independent replicas after evaporation (at 40–50 °C in a water bath using a nitrogen stream) by the initial weight of the sample.

### Instrumental analysis, method validation, and quality assurance

A Trace 1310 GC (Thermo Scientific), coupled to a triple quadrupole MS/MS detector (TSQ 8000 EVO, Thermo Scientific), and an autosampler (Thermo Scientific TriPlusTM) were used for analysis, separation, and confirmation of pesticides and metabolites. Software Xcalibur (version 4.0.27.10, Thermo Scientific) was employed for data acquisition, processing, and overall control of the instrument. Trace Pesticides column (TR-pesticides II, 30 m × 0.25 mm × 0.25 mm + 5 m Guard, REF: 26RD142F) was used for chromatographic separation: initially, set at 80 °C held for 2 min, 20 °C/min to 180 °C, then 5 °C/min rate to 290 °C for 7 min holding time. The injector port temperature was set to 200 °C, and both ion source and MS transfer line were at 290 °C. Helium (99.999% purity) was used at a constant flow rate of 1.2 mL/min as collision gas. Samples (3 µL) were injected in splitless mode (4 mm straight liner, REF: 453A1925), using a 50-mm-long needle; liners were substituted every 200 injections. The ion selection and the collision energies for quantification purposes were obtained through the auto-selected reaction monitoring feature from TSQ dashboard. Fragmentation ions were also compared with other published methods (de Kok et al. 2005; Cruzeiro et al. 2016; EU Reference Laboratories for Residues of Pesticides 2013; Pereira et al. 2014), were used for ion products confirmation and quantification (Table S2).

The validation procedure followed the European Guidance document on Pesticide Residue Analytical Methods SANTE/11813/ 2017 rev 0 (SANTE 2017). Linearity (Table S3) was evaluated using three independent calibration curves, each with six nominal standard concentrations, ranging from 0.06 to 3.84 μg/L (in the final extract or 0.8–25.6 ng/g WW). Curves were plotted using the ratio between the standard and the IS area. The limits of detection (LOD) and quantification (LOQ) were determined, using the following formulas: LOD = 3.3 α/S and LOQ = 10 α/S, where α is the standard deviation of the response and *S* is the average slope of the calibration curves. Recoveries, accuracy, and precision were evaluated by analysing three independent replicates of two different quality control samples (QCs): 2xLOQ and 4xLOQ. Recoveries were carried out on control (blank) samples by spiking the mixture of target compounds at 2xLOQ and 4xLOQ concentrations. Matrix effect (ME) was calculated, at the lowest concentration (LOQ), where matrix samples were spiked after extraction (Astandard in matrix) and compared with those of injected standards (Astandards), as indicated in the following equation:

$$\mathrm{ME}\:=-\left(\left(A_{s\mathrm{tandard}}-A_{s\mathrm{tandard}\;\mathrm{in}\;\mathrm{matrix}}\right)/A_{\mathrm{standard}}\right)\;\ast\;100$$

For quantification purposes, calibration curves were performed using different matrices, sequentially (bivalves → crab → shrimp → fish) according to their lipid content (%) and the pigmentation degree in the extract. Every 20 injections, 5xLOQ was injected as QC together with a control matrix sample and 2 solvent samples, to assure the quality of the run and assess the performance of the GC–MS/MS. In order to decrease background noises from the column, 20–30 blank injections between matrixes were also included in the analyses.

### Data and statistical treatment

Species were grouped by categories, bivalves (*M. meretrix*, *A. subcrenata,* and *P. viridis),* crustaceans *(P. pelagicus, S. serrate,* and *M. ensis),* and fish *(B. pectinirostris)*, and used for evaluation of the compounds’ distribution in each category and to evaluate the potential risks for human health through the consumption of seafood contaminated with OCPs.

For graphical representation, and to avoid underestimation, concentrations of pesticides between the limit of detection (LOD) and the limit of quantification (LOQ) were re-calculated as LOQ/2 (Beal 2001), and values < LOD were not considered in the analysis.

Distribution (%) of the OCPs was calculated by dividing the concentration of individual or grouped compounds by the sum of the total quantified OCPs and multiplying this value by 100. For graphical representation, related compounds were grouped as: HCHs (α + β + γ), DDTs (4,4′-DDT + 4,4′-DDE + 4,4′-DDD), heptachlors (heptachlor + heptachlor epoxide), aldrins (aldrin + endrin + dieldrin), and endosulfans (α + β + endosulfan sulphate).

Spearman rank-order correlations (*r*_s_) were applied to test for relationships between the average pesticide concentrations and lipid content for the studied species. Violin plot representation according to the pesticides’ group concentrations was also included, where besides providing information regarding median (middle dashed line) and quartiles (dot line), it also shows the full distribution of the data (per concentration (length) and per probability of distribution of data (width)). GraphPad Prism version 9.00 was utilized for all statistical analyses. One sample *t*-test (https://www.graphpad.com/quickcalcs/oneSampleT1/) was used to compare the average values, in Tables 1 and 2, with the maximum legal value (10 ng/g WW).

**Table 1:** Concentrations (ng/g WW) and detection frequencies (%) of insecticides in different seafood species acquired from Macao (M) and Hong Kong (HK) markets. (-) represents not available information for the corresponding species and location; *, **, and ***, as *p* < 0.05, *p* < 0.01, and *p* < 0.001, respectively, represent significantly different values above the legal concentration (10 ng/g WW)

Category	Species	Location	Concentrations (ng/g WW)			Detection (%)		
			Range	Average	Median	< LOD	LOD-LOQ	> LOQ
Bivalves	*M. meretrix*	M	6.1–195.5	36.01	12.29	71.88	19.27	8.85
		HK	6.0–6.2	6.08	6.06	81.25	13.02	5.73
	*A. subcrenata*	M	5.0–66.6	22.13	15.09	71.25	21.25	7.50
		HK	-	-	-	-	-	-
	*P. viridis*	M	0.6–3.0	1.575	1.31	81.25	12.50	6.25
		HK	1.1–42.1	23.45^***^	30.33	87.08	8.75	4.17
Crustaceans	*P. pelagicus*	M	1.1–45.7	7.62	3.46	61.76	19.12	19.12
		HK	0.9–155.8	10.90	4.10	60.08	17.65	22.27
	*S. serrata*	M	1.5–49.3	13.06	4.07	61.88	20.63	17.50
		HK	1.3–45.4	10.73	4.55	70.54	18.30	11.16
	*M. ensis*	M	11.4–15–8	13.17^**^	12.75	81.37	13.73	4.90
		HK	3.8–30.2	16.74	16.51	90.76	7.56	1.68
Fish	*B. pectinirostris*	M	2.4–324.3	88.14^*^	23.90	58.82	2.94	38.24
		HK	-	-	-	-	-	-

**Table 2:** Concentration of individual and grouped insecticides (median ± SD) in bivalves, crustaceans, and fish collected from Macao (M) and Hong Kong (HK) markets. (-) indicates compound not detected in the corresponding location/matrix; *, **, and ***, as *p* < 0.05, *p* < 0.01, and *p* < 0.001, respectively, represent significant different values above the legal concentration (10 ng/g WW)

	Bivalves (ng/g WW)		Crustaceans (ng/g WW)		Fish (ng/g WW)
	M	HK	M	HK	M
HCHs	38.6 ± 18.7	37.4 ± 4.1	-	-	23.9 ± 3.2
Aldrins	6.1 ± 0.1^***^	6.1 ± 0.1^***^	1.6 ± 0.2	5.0 ± 2.7	-
Heptachlors	52.5 ± 66.8	-	1.2 ± 2.9	1.6 ± 9.3	99.5 ± 8.7
Fenobucarb	5.9	-	3.3	3.7	222.7 ± 101.6
4,4′-DCBP	20.3 ± 9.9	18.1	12.0 ± 0.6	13.4	-
DDTs	5.9 ± 0.9	-	27.1 ± 14.8	39.4 ± 44.1	53.1 ± 50.2
Endosulfans	-	2.0 ± 0.4	4.1 ± 4.1	4.0 ± 0.6	-
Pyrimethanil	-	-	-	5.0 ± 2.7	2.4
Pirimicarb	-	-	2.8	2.8 ± 1.3	3.9 ± 0.07
Methoxychlor	-	-	-	-	9.9 ± 0.3

The complete data set obtained in this work is available in the OSF repository, considered as data management software for open science: https://osf.io/smzbk/?view_only=c5e01507620e4d8ab70c1563b608bda1.

### Human hazard risk assessment

Dietary intake risk of pesticides through seafood consumption was estimated using well-established hazard quotients and indices determined by the US Environmental Protection Agency (EPA 1998) and European Food Safety Authority (EFSA, 2014). In this study, we studied the carcinogenic and non-carcinogenic risks for adults and toddlers consuming seafood acquired from these markets.

The average concentration values were used for the evaluation of the human health risk assessment since they tend to be higher than the median ones, after which a worst-case scenario was assumed.

#### Non-carcinogenic risk

For short-term or acute exposure (1–7 days): the estimated maximum daily intake (EMDI) was determined by multiplying the maximum compound value quantified in the matrix (mg/kg of WW) by the consumption rate (kg/capita/day), and then divided by the bodyweight (kg) (Chinese adult (63 kg) and toddler (14 kg) (Xiao et al. 2018; Hu et al. 2011). The acute reference dose (ARfD), an estimation of the amount of substance in food that can be ingested over a short period, was gathered from the “Pesticides Properties Data Base” website (PPDB, 2021), and the hazard quotient was calculated as follows:

$${\mathrm{HQ}}_{\mathrm{ST}}=\mathrm{EMDI}/\mathrm{ARfD}$$

For long-term or chronic exposure (> 21 days): the estimated average daily intake (EADI) was calculated as mentioned above but using the average compound value quantified in the matrix (mg/kg of WW). According to the US Environmental Protection Agency, the EADI of pesticide residues should be less than the established acceptable daily intake (ADI), which estimates the amount of a substance in food that can be ingested daily over a lifetime without appreciable health risk to the consumer (EPA, 1998). Values of ADI were obtained from the PPDB (2021), and the hazard quotient was calculated as follows:

$${\mathrm{HQ}}_{\mathrm{LT}}=\mathrm{EADI}/\mathrm{ADI}$$

Although these HQs are calculated considering different assumptions, all of them measure the development of potential non-carcinogenic health risk effects (Cruzeiro et al. 2018; Alsafran et al. 2021; Ju et al. 2022).

For both short- and long-term risk assessments, average daily consumption of 17.1 g/person/day (bivalves), 8.3 g/person/day (crustaceans), and 105 g/person/day (fish) were used as described by Liu et al. (2018), for coastal Chinese populations. For toddlers, only the average fish consumption (50 g/toddler/day) was used, since there is no official information regarding the consumption of molluscs and crustaceans for toddlers (EFSA Dietetic Products, Nutrition, and Allergies (NDA) 2014).

Finally, in order to consider the cumulative risk assessment, the health risk index (HI) was calculated as the sum of insecticides (ΣHQ_LT_). If HI < 1, there is no apparent health impact; if HI > 1.0, there is the possibility of an adverse health effect (Salam et al. 2021).

#### Carcinogenic risk

Evaluation of the cancer risk (CR) presented to human health by accidental ingestion of potential carcinogenic insecticides was calculated by multiplying the estimated average daily intake (EADI; mg/day/kg body weight) by the cancer slope factor of the compound (CSF; mg/kg/day) (Alsafran et al. 2021; Bodor et al. 2021):

$$\mathrm{CR}=\mathrm{EADI}\times \mathrm{CSF}$$

The CSF was obtained from the Office of Environmental Health Hazard Assessment (OEHHA, 2021), and from other published works (Jiang et al. 2005; Akoto et al. 2015). The cumulative cancer risk (TCR), was calculated as (Alsafran et al. 2021):

$$\mathrm{TCR}=\mathrm{\Sigma CR}$$

If the cumulative values are within the range 10^–6^–10^–4^, admissible cancer risk levels are expected, which means that the probability for cancer development during a human lifetime (70 years) is 1/1,000,000, or 1/10,000, respectively. Generally speaking, a cumulative cancer risk higher than 10^−4^ is not accepted, and the maximum tolerable value is 10^−5^ (Bodor et al. 2021).

## Results

### QuEChERS method validation

During validation processes, endrin aldehyde and endrin ketone were excluded due to poor signal intensity, at least within the target range of concentrations (1.2–76.8 ng/g). 4,4′-DDD and 4,4′-DDE were overlapping; therefore, quantification was done as the combination of both to avoid overestimation. Due to dicofol instability (highly hydrolysable compound), we quantified its main metabolite (4,4′-DCBP) as already done in previous works with this compound (Ivorra et al. 2019a, b). Regarding the other compounds, all the validation criteria were successfully established for all the selected matrices (bivalves, crabs, shrimp, and fish), proving that the extraction and chromatographic method is robust enough to quantify the rest of the 18 pesticides.

The calibration curves had good fits, with *r*^2^ ranging from 0.802 to 0.999. LODs were below 2.52 ng/g WW, 0.81 ng/g WW, 0.96 ng/g WW, and 1.49 ng/g WW for bivalves, crab, shrimp, and fish matrix, respectively; the LOQs ranged from 0.05 to 7.65 ng/g WW, 0.03 to 2.46 ng/g WW, 0.17 to 2.91 ng/g WW, and 0.17 to 2.91 WW ng/g for bivalves, crab, shrimp, and fish matrix, respectively.

The quantified concentrations accomplished the limits established (10 ng/g WW) set in the annex IIA point 4.2.1 of 91/414/EEC established by the European Commission Directorate General Health and Consumer Protection (SANCO, 2010) and the regulatory levels for marine biological quality enacted by the Chinese Government (GB184-2001). Detailed information regarding LODs, LOQs for each compound, and matrix can be found in Table S4 (supplementary material).

Recovery, precision, and accuracy tests were done in the bivalves’ matrix and details are described in Table S5 (supplementary material). Regarding the matrix effect, 39% of the compounds presented signal enhancement, while 61% had signal suppression in the matrix. Matrix-matched calibration curves were used to avoid quantification deviations due to the matrix effect. Detailed information for each compound can be found in Fig S3 (supplementary material). To guarantee the stability of the extracts, samples were injected over a 24-h period.

### Insecticides values in edible species

Quantified OCPs in each species plus their percentage of detection are listed in Table 1 and grouped by categories (bivalves, crustaceans, and fish). Additionally, information regarding lipid content in each category, which was not significantly different (*p* < 0.05), and the concentration of insecticide expressed per lipid content can be found in the supplementary material (Table S6, Fig. S3 and S4, respectively). All the validated compounds, except for lindane (γ-HCH), were quantified in the 138 seafood samples. Mussels (*P. viridis*) collected from Hong Kong markets, together with mudskippers (*B. pectinirostris*), from Macao presented the highest OCP concentrations with median values of 30.33 and 23.90 ng/g WW, respectively. In both cases, the quantified concentrations were significantly higher (*p* < 0.05) than the established legal threshold (10 ng/g WW). Mudskippers were also the animals with the highest frequency of quantification of the target compounds (38%). On the other hand, crabs (*P. pelagicus* and *S. serrata*), acquired from Macao and Hong Kong markets, presented the lowest OCPs levels (ca. 4 ng/g WW), with detection frequencies ranging from 11 to 22%. The shrimp *M. ensis* presented median values of 14.63 ng/g WW but with the lowest detection rates (< 5%). Average concentrations were also significantly different (*p* < 0.05) from the target 10 ng/g WW in shrimps bought in Macau.

**Fig. 1:**
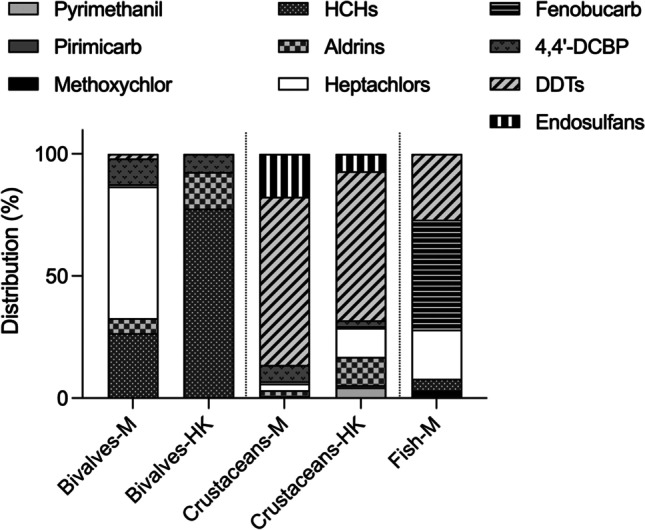
Distribution (normalized %) of individual and grouped OCPs by categories (bivalves, crustaceans, and fish) and location (Macao, M; Hong Kong, HK)

The relative distribution of OCPs and concentrations quantified in each category, per market’s location, are shown in Fig. 1 and Table 2, respectively.

Interestingly, the relative percentage of compounds differs among matrices but also within, being more evident for bivalves acquired in Macao markets, due to the high heptachlors’ median concentration (52.55 ng/g WW) quantified in this matrix. This compound was also the most representative one (54%) for this matrix and location. Overall, HCH median levels (ca. 38 ng/g WW, in both sites) and frequencies (27% and 78% for Macao and Hong Kong, respectively) were higher in this matrix when compared to crustaceans or fish. In crustaceans, DDTs were the most representative ones (ca. 65%) for both Macao and Hong Kong markets, with median values of 27.06 and 39.38 ng/g WW, respectively. In fish, heptachlors-DDTs-fenobucarb presented a more homogenous distribution (20–27-45%), with median concentrations of 99.50–53.06–222.66 ng/g WW, respectively. For this category, no comparison between sites could be done due to the lack of available mudskippers from Hong Kong markets.

Correlations between lipid content and OCPs quantified in bivalves, crustaceans, and fish were not significant (*r*^2^ = 0.029; *p* ≥ 0.05). Detailed data regarding lipid content quantified for each species is included in the supplementary data (Table S6).

### Human risk assessment and EU values

Parameters like consumption rates of bivalves, crustaceans and fish, EADI, ADI, EDI, CSF, and ARfD values, were used to quantify the potential non-carcinogenic and carcinogenic risk, and are summarized in Tables 3 and 4, respectively; detailed results can be found in Tables S7–S14 (supplementary material).

**Table 3:** Hazard quotients (HQs) considering long-term (LT) and short-term (ST) exposure to pollutants through consumption, and hazard index (HI) of the insecticide’s concentrations quantified in fish, bivalves, and crustaceans from Hong Kong and Macao markets. *EMDI* estimated maximum daily intake; *EADI* estimated average daily intake; *ADI* acceptable daily intake; *ArfD* acute reference dose; (^) means compound not detected; (*) means ArfD/ADI not available; data related to toddlers is represented with the symbol (#); HQ_LT_>1 values are in bold

	HQ_ST_ = EMDI/ArfD				HQ_LT_ = EADI/ADI			
	Fish#	Fish	Bivalves	Crustaceans	Fish*	Fish	Bivalves	Crustaceans
∑aldrin + dieldrin	^	^	3.77E-03	4.59E-03	^	^	7.45E-02	7.37E-02
Fenobucarb	*	*	*	*	1.32E-02	6.74E-03	5.36E-05	1.05E-04
DDTs	*	*	*	*	4.67E-02	2.18E-02	1.93E-03	1.33E-02
4,4’-DCBP	*^	*^	*	*	*^	*^	*	*
Endosulfans	^	^	^	4.77E-04	^	^	^	1.27E-03
Methoxychlor	7.31E-03	3.41E-03	6.53E-04	2.13E-03	3.54E-04	1.65E-04	2.13E-05	1.07E-04
Heptachlor	*^	*^	*^	*	^	^	^	1.77E-02
HCH (α)	^	^	^	^	^	^	^	^
HCH (β)	1.86E-02	8.70E-03	2.63E-02	^	1.51E-02	7.04E-03	9.37E-03	^
HCH (γ)	^	^	^	^	^	^	^	^
Hept. epoxide	*	*	*	*	**3.45E + 00**	**1.61E + 00**	9.13E-01	1.53E-01
Endrin	^	^	6.72E-03	1.56E-02	^	^	5.33E-03	1.02E-02
Pirimicarb	1.41E-04	6.60E-05	6.83E-06	6.84E-05	3.95E-04	1.84E-04	1.90E-05	5.63E-05
Pyrimethanil	^	^	2.16E-07	1.24E-05	^	^	1.08E-06	2.49E-05
HI:	2.61E-02	1.22E-02	3.75E-02	2.29E-02	**3.52E + 00**	**1.65E + 00**	**1.00E + 00**	2.69E-01

**Table 4:** Cancer risk (CR), and total cancer risk (TCR) of insecticide concentrations quantified in fish, bivalves, and crustaceans from Hong Kong and Macao markets. *EADI* estimated daily intake; *CSF* cancer slope factor; (^) means compound not detected; (*) means CSF not available; (/) means compound not considered as carcinogenic; # symbol means data related to toddlers; CR > E−4 are in bold

Compound	CR = EADI * CSF			
	Fish#	Fish	Bivalves	Crustacean
∑aldrin + dieldrin	^	^	**1.20E-04**	**1.18E-04**
Fenobucarb	*	*	*	*
DDTs	7.77E-05	3.74E-05	3.26E-06	2.24E-05
4,4′-DCBP	* ^	* ^	*	*
Endosulfans	/	/	/	/
Methoxychlor	/	/	/	/
Heptachlor	^	^	^	8.10E-06
HCH (α)	*^	*^	*^	*^
HCH (β)	**1.56E-04**	6.30E-05	8.46E-05	^
HCH (γ)	^	^	^	^
Hept. epoxide	**1.92E-03**	**8.80E-04**	**5.01E-04**	8.25E-05
Endrin	/	/	/	/
Pirimicarb	*	*	*	*
Pyrimethanil	*^	*^	*	*
TCR	**2.15E-03**	**9.80E-04**	**7.08E-04**	**2.31E-04**

#### Non-carcinogenic risk

Acute exposure risk assessment (HQ_ST_) cannot be done for fenobucarb, DDTs, heptachlor, heptachlor epoxide, and 4,4′-DCBP because there were no available ARfD data. For the rest of the compounds, HQ_ST_ < 1 for all the categories (bivalves, crustaceans, and fish).

For chronic exposure risk assessment (HQ_LT_), only heptachlor epoxide (96.91 ng/g WW) presented a HQ_LT_ > 1, in fish, for both adults and toddlers.

In this study, all the seafood samples contained multiple pesticide residues, and results regarding the cumulative risk presented HI > 1 for fish and bivalves.

#### Carcinogenic risk

Compounds such as endosulfans, methoxychlor, and endrin were not included in this calculation, since they are not classified as carcinogenic compounds to humans according to US EPA (Group D) (IRIS 2021). Also, fenobucarb, α-HCH, 4,4′-DCBP, pirimicarb, and pyrimethanil were not included because there is no CSF information available for these.

All CR values are inside the potentially dangerous range previously indicated (10^–4^–10^–6^), where results above 10^−4^ (highlighted in bold in Table 4) indicate that consumption of the seafood studied may induce cancer risk to adults and toddlers. Additionally, and as expected due to the individual results obtained, TCR values were also above the threshold value (> 10^−4^) for all the matrices (Table 4).

#### Legislation values

**Fig. 2:**
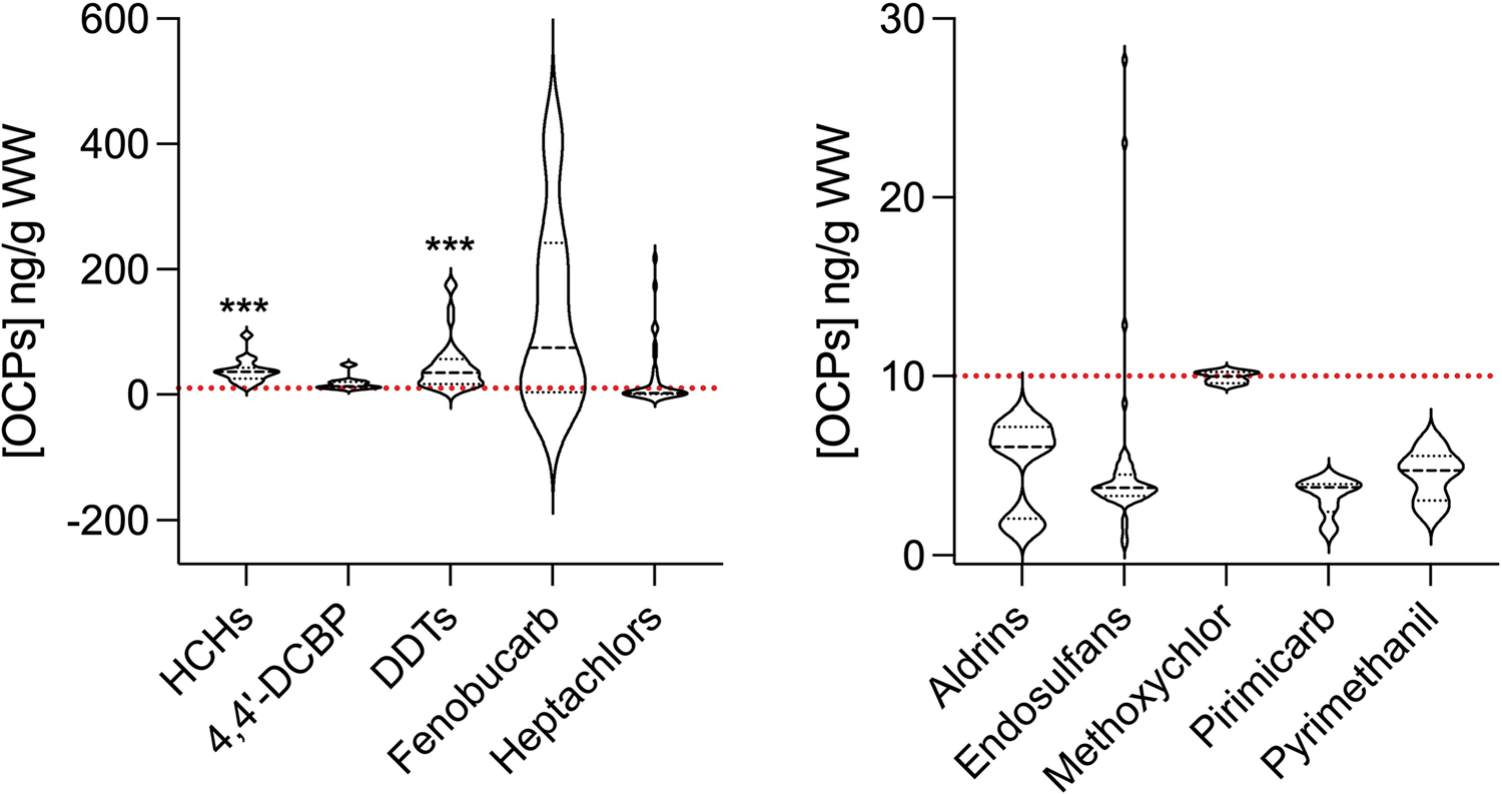
Distribution of grouped OCPS concentrations (ng/g WW) considering the seafood samples acquired from both market regions. The red line indicates the threshold limit (10 ng/g WW); (*, **, and ***, as *p* < 0.05, *p* < 0.01, and *p* < 0.001, respectively, represent significantly different values above the legal concentration (10 ng/g WW)

The median concentrations of the quantified compounds were compared against the maximum admissible concentration (10 ng/g WW) established by the regulatory agencies described above. Results, summarized in Fig. 2, showed that median values of fenobucarb, DDTs, and HCHs median values were 13-, 4.6- and 3.8-fold above this limit, respectively, being significantly different (*p* < 0.0001) from this threshold.

## Discussion

### Occurrence of insecticides on seafood

Among the quantified compounds, DDTs + fenobucarb and HCHs + heptachlors stand out, in fish and bivalves’ matrix, respectively, due to their high concentrations and their high frequencies of detection. However, no significant correlations between lipid content and the quantified insecticides were found, as expected in H1, which may be due to no significant differences in lipid content between categories.

The availability and distribution of organic chemicals in the environment (habitat pollution), and between organisms, is a complex process. Factors such as bioavailability, solubility, sorption, uptake, metabolism, retention, release, or excretion of the organic chemical are fundamental for this process (Van der Oost et al. 2003, Tsygankov 2019; Hou et al. 2021). In addition, biodegradation or biotransformation of some compounds might influence the bioaccumulation of organochlorinated compounds, among different species (Cappelletti et al. 2015). In this case, mudskippers (here representing the fish category) can accumulate contaminants via two different routes: bioconcentration (dermal absorption of contaminants) and bioaccumulation (assimilation of contaminants by food intake) (Ansari et al. 2014). A similar pattern was also observed and highlighted in our previous work (Ivorra et al. 2021), which can explain the high levels quantified in these organisms when compared to the other categories. This was also explored by Fan et al. (2019) when quantifying several endocrine disruptors in fish with different feeding habits.

Within the quantified compounds in fish, fenobucarb (followed by DDTs) was quantified at higher concentrations. As it was previously mentioned, this insecticide is still allowed and currently being used in China and is the only one considered as a non-banned insecticide (Lan et al. 2019) within the target compounds included in this study. Zheng et al. (2016), considered fenobucarb as one of the most dominant pesticides quantified in estuarine waters and sediments from Jiulong River (Fujian province), where concentrations varied seasonally, being highly distributed during summertime (July), matching with our sampling season (June–August). Moreover, Xu et al. (2019) quantified this compound in 92% of the samples collected from different watersheds across China. Another study showed that fenobucarb (together with isoprocarb) presented the highest ecological risk, among 29 pesticides quantified in Huangpu River (Shanghai) waters (Xu, et al. 2020).

In addition, DDT levels quantified in mudskippers from Macao were also relevant, although this compound has been already banned since the 1980s (Guo et al. 2009). Surprisingly, DDTs were quantified at higher concentrations in this study than in previous monitoring reports (Lam and Lam 2004, Wong et al. 2005). For example, mudskippers (*B. pectinirostris* and *B. boddarti*) collected from Mai Po areas in Hong Kong, presented 13- and twofold lower than the values quantified in the present work, suggesting that DDT pollution in the south China area is still a current concern (Guo et al. 2009; Ivorra et al. 2021).

Regarding bivalves, HCHs and heptachlors were the most representative ones. Due to their higher-water solubility (log k_ow_ 3.5 and 4.8, respectively), these compounds tend to be in the aqueous phase. According to Yang et al. (2014), higher levels of these pesticides were found in estuarine water when compared to more hydrophobic ones, such as DDTs (log k_ow_ 6.9). Also, as filter-feeders, bivalves capture their food through filtration (dissolved and particulate matter), and therefore, they can be more susceptible to the uptake of these pesticides. Both compounds were quantified, in Hong Kong and Macao markets, at superior levels (38.2–53.4 ng/g WW) than other Chinese (0.5–0.9 ng/g WW) and European (0.1–0.3 ng/g WW) markets (Binelli and Provini 2003; Yang et al. 2006; Guo et al. 2007; Liu et al. 2010). This probably indicates that animals were collected from polluted areas, in the south of China and Southeast Asia. It is well known that this region is highly impacted by different pollutants and several studies (Hu et al. 2016; Li et al. 2021a, b; Wang et al. 2022) already addressed their concern about seafood quality for human consumption.

Overall, crustaceans presented lower levels of insecticides than fish or bivalves (7.3- and 1.8-fold, respectively), although, within the different compounds, DTTs stand out with concentrations of ca. 33 ng/g WW. The same range of concentrations (ca. 30 ng/g WW) were reported by Chen et al. (2022), from several aquaculture ponds in China. This indicates that DDT is still detected in commercial samples, reflecting the need for active monitoring programs in market samples. DDT levels quantified here were also higher than from crustaceans collected in other Asian countries (20-fold higher than in Bangladesh or Singapore (Bayen et al. 2005; Borrell et al. 2019), or even 40-fold higher than in Taiwan (Das et al. 2020).

### Human risk assessment and legislation values

According to Carvalho (2017), fish and seafood consumption is considered one of the main pathways of human exposure to organic contaminants. Based on the values quantified in this work, we performed a human risk assessment (for toddler and adult consumers).

Evaluation of non-carcinogenic risks revealed no acute risk (short-term) associated with the consumption of the selected animals. However, we cannot neglect the potential health risk associated with seafood consumption where pesticides like DDTs and, HCHs, fenobucarb, and heptachlors were detected at levels above the maximum admissible concentrations suggested by the legislation discussed above (SANCO 2010; GB184-2001). Besides, this assessment is quite conservative since the acute reference dose for several pesticides and metabolites (e.g., fenobucarb, DDTs, heptachlors, 4,4′-DCBP) are not available, limiting the analyses.

Additionally, we also observe a potential chronic health effect for the fish matrix and a cumulative risk for fish and bivalves’ matrices, which is in partial agreement with H2. These results may indicate that the consumption of fish (mudskippers) and bivalves from Southeast China could have a potential threat to human health. Guo et al. (2009) had analogous results where DDT was quantified in several seafood samples from markets in Guangdong province, indicating that consumption of bivalves (i.e., clams, mussels, and oysters) and fish (i.e., tilapia, grass carp, Chinese perch) may possess higher concerns than crustaceans (shrimp and crabs).

Results from our study also revealed that pesticides residues quantified in seafood samples (fish and bivalves) from Hong Kong and Macao markets may have a carcinogenic risk (including to toddlers that consume fish). Jiang et al. (2005) reached similar results with fish acquired from Zhousan (China) markets. These authors concluded that fish consumption had a greater lifetime cancer risk than one in one million (< 10^–6^) and highlighting DDTs as a pesticide with a particular concern. Similar results were also obtained by Guo et al. (2007), who indicated a potential health risk associated with the consumption of seafood products coming from southern China, highlighting also DDTs as a special concern. The consumption of seafood by Chinese citizens continues to rise (i.e., from 27.1 g/day in 1992, 30.1 g/day in 2002, to 70.3 g/day in 2011) (Zhang et al. 2012; Wang et al. 2015). However, the high levels of pesticides and other compounds found along the Chinese coasts (Guo et al. 2008a, b; Hu et al. 2016; Min et al. 2020; He et al. 2021; Li et al. 2021a, b; Wang et al. 2022) are compromising the final quality of the seafood products, which in turn can compromise the human health (adults and children) and also other top-chain predators (not explored here).

## Conclusion

The quantification of insecticides and some metabolites was performed in commercial seafood samples acquired from Macao and Hong Kong markets, together with their potential health risk evaluation. Within the 7 selected species, the bivalve *P. viridis* (Hong Kong market) and the mudskipper *B. pectinirostris* (Macao market) presented the highest concentrations. Nevertheless, it is important to consider that accumulation of insecticides by organisms is probably related to the status of the surrounding ecosystem (South China), which in this case is represented by a vast marine and coastal area. Residual levels of fenobucarb, DDTs, HCHs, and heptachlors were the most concerning ones, demonstrating that some banned compounds can still be detected in the environment and could compromise the health status of the consumers since the health risk assessment indicated that bivalves and mudskippers could present a potential threat for human consumption (adult and toddlers), and could raise concerns of lifetime cancer development. This health risk evaluation of pesticide residues was done considering the intake of a single matrix; however, the diet of any Chinese adult includes different animal products, which increases the probability of a higher hazardous risk. Besides, the lack of information regarding some compounds and metabolites (acute reference dose and/or the acceptable daily intake values) limits the analyses. Since the ultimate accumulator of chemical pollutants is the human body, this can lead to health problems. Therefore, we suggest that risk management aimed at controlling human exposure to OCPs should be done. This can be better achieved if the seafood sources (i.e., aquacultures and/or wild coastal environments) are rigorously controlled.

## Supplementary Information

Below is the link to the electronic supplementary material.

Supplementary file1 (DOCX 4298 KB)

## Data Availability

The datasets generated during the current study are available in the OSF repository, https://osf.io/smzbk/?view_only=c5e01507620e4d8ab70c1563b608bda1. The authors confirm that the data supporting the findings of this study are available within the article [and/or] its supplementary materials.

## Abbreviations

*ADI*: Established acceptable daily intake
*AEDI*: Estimated average daily intake
*ARfD*: Acute reference dose
*CR*: Cancer risk
*CSF*: Cancer slope factor of the compound
*DCBP*: Dichlorobenzophenone
*DDD*: Dichlorodiphenyldichloroethane
*DDE*: Dichlorodiphenyldichloroethylene
*DDTs*: Dichlorodiphenyltrichloroethane
*EMDI*: Estimated maximum daily intake
*HCHs*: Hexachlorocyclohexane
*HI*: Health risk index
*HQ*_*LT*_: Hazard quotient long term
*HQ*_*ST*_: Hazard quotient short term
*LOD*: Limit of detection
*log K*_*ow*_: Octanol water partition coefficient
*LOQ*: Limit of quantification
*OCPs*: Organochlorine pesticides
*PRD*: Pearl River Delta
*TCR*: Cumulative cancer risk
*WW*: Wet weight

## References

[CR1] AFCD (2021) Agriculture, Fisheries and Conservation Department from Hong Kong. https://www.afcd.gov.hk/english/fisheries/fish_aqu/fish_aqu_mpo/fish_aqu_mpo.html (Accessed, July 29 2021)

[CR2] Akoto O, Oppong-Otoo J, Osei-Fosu P (2015). Carcinogenic and non-carcinogenic risk of organochlorine pesticide residues in processed cereal-based complementary foods for infants and young children in Ghana. Chemosphere.

[CR3] Alsafran M, Usman K, Rizwan M, Ahmed T, Al Jabri H (2021). The carcinogenic and non-carcinogenic health risks of metalloids bioaccumulation in leafy vegetables: a consumption advisory. Front Environ Sci.

[CR4] Ansari AA, Trivedi S, Saggu S, Rebman H (2014). Mudskipper: a biological indicator for environmental monitoring and assessment of coastal waters. Journal of Entomology and Zoology Studies.

[CR5] Bayen S, Wurl O, Karuppiah S, Sivasothi N, Lee HK, Obbard JP (2005). Persistent organic pollutants in mangrove food webs in Singapore. Chemosphere.

[CR6] Beal SJ (2001). Ways to fit a PK model with some data below the quantification limit. J Pharmacokinet Pharmacodyn.

[CR7] Binelli A, Provini A (2003). POPs in edible clams from different Italian and European markets and possible human health risk. Mar Pollut Bull.

[CR8] Bodor K, Bodor Z, Szép A, Szép R (2021). Human health impact assessment and temporal distribution of trace elements in Copșa Mică- Romania. Sci Rep.

[CR9] Borrell A, Tornero V, Bhattacharjee D, Aguilar A (2019). Organochlorine concentrations in aquatic organisms from different trophic levels of the Sundarbans mangrove ecosystem and their implications for human consumption. Environ Pollut.

[CR10] Cappelletti N, Speranza E, Tatone L, Astoviza M, Migoya MC, Colombo JC (2015). Bioaccumulation of dioxin-like PCBs and PBDEs by detritus-feeding fish in the Rio de la Plata estuary, Argentina. Environ Sci Pollut Res.

[CR11] Carvalho FP (2017). Pesticides, environment, and food safety. Food Energy Secur.

[CR12] Chen J, Guang C, Xu H, Chen Z, Xu P, Yan X, Wang Y, Liu J (2008) Marine fish cage culture in China. In A. Lovatelli, M.J. Phillips, J.R. Arthur and K. Yamamoto (eds). FAO/NACA Regional Workshop on the Future of Mariculture: a Regional Approach for Responsible Development in the Asia-Pacific Region. Guangzhou, China, 7–11 March 2006. FAO Fisheries Proceedings. No. 11. Rome, FAO. 2008. pp. 285–299.

[CR13] Chen L, Qian Y, Jia Q, Weng R, Zhang X, Li Y, Qiu J (2022) A large geographic-scale characterization of organochlorine pesticides (OCPs) in surface sediments and multiple aquatic foods of inland freshwater aquaculture ponds in China: Co-occurrence, source and risk assessment. Environ Pollut 308:119716. 10.1016/j.envpol.2022.119716 10.1016/j.envpol.2022.119716 35809714

[CR14] Cruzeiro C, Rodrigues-Oliveira N, Velhote S, Pardal MA, Rocha E, Rocha MJ (2016). Development and application of a QuEChERS-based extraction method for the analysis of 55 pesticides in the bivalve *Scrobicularia plana* by GC-MS/MS. Anal Bioanal Chem.

[CR15] Cruzeiro C, Rocha E, Rocha MJ (2018) Pesticides in worldwide aquatic systems: Part II. Estuary, William Froneman. IntechOpen.10.5772/intechopen.73117

[CR16] Das S, Aria A, Cheng JO, Souissi S, Hwang JS, Ko FC (2020). Occurrence and distribution of anthropogenic persistent organic pollutants in coastal sediments and mud shrimps from the wetland of central Taiwan. PLoS One.

[CR17] de Kok A, Hiemstra M, van Bodegraven P (2005) Validation of a fast and easy method for the determination of residues from 229 pesticides in fruits and vegetables using gas and liquid chromatography and mass spectrometric detection. J AOAC Int 88:595–614. 10.1093/jaoac/88.2.595 15859089

[CR18] EFSA Dietetic Products, Nutrition, and Allergies (NDA) (2014) Scientific Opinion on health benefits of seafood (fish and shellfish) consumption in relation to health risks associated with exposure to methylmercury. EFSA J 12(7):3761

[CR19] EU Reference Laboratories for Residues of Pesticides (2013) Analysis of dicofol via QuECHERS - use of isotope labeled dicofol to improve precision 1–6

[CR20] European Commission Directorate General Health and Consumer Protection (2010) Guidance document on pesticides residue analytical methods. In: Directorate General Health and Consumer Protection. SANCO/825/00 rev 8.1

[CR21] European Commission Directorate General Health and Consumer Protection (2017) Guidance Document on Analytical Quality Control and Method Validation Procedures for Pesticide Residues and Analysis in Food and Feed. SANTE/11013/2017 rev 0

[CR22] Fan JJ, Wang S, Tang JP, Zhao JL, Wang L, Wang JX, Liu SL, Li F, Long SX, Yang Y (2019). Bioaccumulation of endocrine disrupting compounds in fish with different feeding habits along the largest subtropical river, China. Environ Pollut.

[CR23] FAO (2018) Food and Agriculture Organization of the United Nations. http://www.fao.org/faostat/en/#data/FBS. Accessed 11 Jan 2021

[CR24] GB 2763-2014 (2014) National food safety standard — maximum residue limits for pesticides in food, national health and family planning commission of the People’s Republic of China and Ministry of Agriculture of the People’s Republic of China (translated version). https://www.chinesestandard.net/PDF/BOOK.aspx/GB2763-2014

[CR25] Grung M, Lin Y, Zhang H, Steen AO, Huang J, Zhang G, Larssen T (2015). Pesticide levels and environmental risk in aquatic environments in China — A review. Environ Int.

[CR26] Guo JY, Zeng EY, Wu FC, Meng XZ, Mai BX, Luo XJ (2007). Organochlorine pesticides in seafood products from southern China and health risk assessment. Environ Toxicol Chem.

[CR27] Guo Y, Meng XZ, Tang HL, Zeng EY (2008). Tissue distribution of organochlorine pesticides in fish collected from the Pearl River Delta, China: implications for fishery input source and bioaccumulation. Environ Pollut.

[CR28] Guo L, Qiu Y, Zhang G, Zheng GJ, Lam PKS, Li X (2008). Levels and bioaccumulation of organochlorine pesticides (OCPs) and polybrominated diphenyl ethers (PBDEs) in fishes from the Pearl River estuary and Daya Bay, South China. Environ Pollut.

[CR29] Guo Y, Yu HY, Zeng EY (2009). Occurrence, source diagnosis, and biological effect assessment of DDT and its metabolites in various environmental compartments of the Pearl River Delta, South China: a review. Environ Pollut.

[CR30] He Y, Guo C, Lv J, Deng Y, Xu J (2021) Occurrence, sources, and ecological risks of three classes of insecticides in sediments of the Liaohe River basin, China. Environ Sci Pollut Res 28:62726–62735. 10.1007/s11356-021-15060-5 10.1007/s11356-021-15060-5 34212336

[CR31] Hou R, Lin L, Li H, Liu S, Xu X, Xu Y, Jin X, Yuan Y, Wang Z (2021). Occurrence, bioaccumulation, fate, and risk assessment of novel brominated flame retardants (NBFRs) in aquatic environments—A critical review. Water Res.

[CR32] Hu S, Su Z, Jiang J, Huang W, Liang X, Hu J, Chen M, Cai W, Wang J, Zhang X (2016). Lead, cadmium pollution of seafood and human health risk assessment in the coastline of the southern China. Stoch Env Res Risk Assess.

[CR33] Hu Y, Qi S, Zhang J, Tan L, Zhang J, Wang Y, Yuan D (2011). Assessment of organochlorine pesticides contamination in underground rivers in Chongqing, Southwest China. J Geochem Explor.

[CR34] IRIS : Integrated Risk Information System (2021) US EPA. https://cfpub.epa.gov/ncea/iris_drafts/simple_list.cfm?list_type=pesticide. (Accessed, 25 October 2021)

[CR35] Ivorra L, Cardoso PG, Chan SK, Tagulao K, Cruzeiro C (2019a) Environmental characterization of 4, 4′-dichlorobenzophenone in surface waters from Macao and Hong Kong coastal areas (Pearl River Delta) and its toxicity on two biological models: *Artemia salina *and *Daphnia magna*. Ecotoxicol Environ Saf 171:1–11. 10.1016/j.ecoenv.2018.12.054 10.1016/j.ecoenv.2018.12.054 30583221

[CR36] Ivorra L, Cruzeiro C, Chan SK, Tagulao KA, Cardoso PG (2019b) Uptake and depuration kinetics of dicofol metabolite 4, 4′-dichlorobenzophenone, in the edible Asiatic clam *Meretrix meretrix*. Chemosphere 235:662–669. 10.1016/j.chemosphere.2019.06.155 10.1016/j.chemosphere.2019.06.155 31276879

[CR37] Ivorra L, Cardoso PG, Chan SK, Cruzeiro C, Tagulao KA (2021). Can mangroves work as an effective phytoremediation tool for pesticide contamination? An interlinked analysis between surface water, sediments and biota. J Clean Prod.

[CR38] Jiang QT, Lee TKM, Chen K, Wong HL, Zheng JS, Giesy JP, Lo KKW, Yamashita N, Lam PKS (2005). Human health risk assessment of organochlorines associated with fish consumption in a coastal city in China. Environ Pollut.

[CR39] Ju YR, Chen CF, Wang MH, Chen CW, Dong CD (2022). Assessment of polycyclic aromatic hydrocarbons in seafood collected from coastal aquaculture ponds in Taiwan and human health risk assessment. J Hazard Mater.

[CR40] Lam PKS, Lam MHW (2004) Assessment of risk to the Mai Po/Inner Deep Bay Ramsar site due to environmental contaminants. In Developments in Ecosystems, volume 1, chapter 8, pp. 115–129. 10.1016/S1572-7785(04)01008-1

[CR41] Lan J, Jia J, Liu A, Yu Z, Zhao Z (2019). Pollution levels of banned and non-banned pesticides in surface sediments from the East China Sea. Mar Pollut Bull.

[CR42] Li Y, Guo N, Zou X, Li P, Zou S, Luo J, Yang Y (2021). Pollution level and health risk assessment of polycyclic aromatic hydrocarbons in marine fish from two coastal regions, the South China Sea. Mar Pollut Bull.

[CR43] Li H, Jiang W, Pan Y, Li F, Wang C, Tian H (2021). Occurrence and partition of organochlorine pesticides (OCPs) in water, sediment, and organisms from the eastern sea area of Shandong Peninsula, Yellow Sea. China Marine Pollution Bulletin.

[CR44] Liu Z, Zhang H, Tao M, Yang S, Wang L, Liu Y, Ma D, He Z (2010). Organochlorine Pesticides in Consumer Fish and Mollusks of Liaoning Province, China: Distribution and Human Exposure Implications. Arch Environ Contam Toxicol.

[CR45] Liu WX, Hou JY, Wang QL, Yang HJ, Luo YM, Christie P (2015) Collection and analysis of root exudates of *Festuca arundinacea* L. and their role in facilitating the phytoremediation of petroleum- contaminated soil. Plant Soil 389:109–119. 10.1007/s11104-014-2345-9

[CR46] Liu Q, Liao Y, Shou L (2018). Concentration and potential health risk of heavy metals in seafoods collected from Sanmen Bay and its adjacent areas, China. Mar Pollut Bull.

[CR47] Monirith I, Ueno D, Takahashi S, Nakata H, Sudaryanto A, Subramanian A, Karuppiah S, Ismail A, Muchtar M, Zheng JJ, Richardson B, Prudente M, Due Hue N, Seang Tana TV, Tkalin A, Tanabe S (2003). Asia-Pacific mussel watch: monitoring contamination of persistent organochlorine compounds in coastal waters of Asian countries. Mar Pollut Bull.

[CR48] Min L, Xiaolin L, Xiaotong G, Chunyang L, Dufa G, Jing M, Xiaqing W, Ruichen Z, Dongyan L, Dongqi W, Yanchuang Z, Lingxin C (2020). A national-scale characterization of organochlorine pesticides (OCPs) in intertidal sediment of China: Occurrence, fate and influential factors. Environ Pollut.

[CR49] Nakata H, Kawazoe M, Arizono K, Abe S, Kitano T, Shimada H, Li W, Ding X (2002). Organochlorine Pesticides and Polychlorinated Biphenyl Residues in Foodstuffs and Human Tissues from China: Status of Contamination, Historical Trend, and Human Dietary Exposure. Arch Environ Contam Toxicol.

[CR50] O´Connor TP (2002) National distribution of chemical concentrations in mussels and oysters in the USA. Marine Environmental Research. 53, 117–143. OSPAR., 2002. 10.1016/s0141-1136(01)00116-7 10.1016/s0141-1136(01)00116-7 11824825

[CR51] OEHHA : Office of Environmental Health Hazard Assessment (2021) http://oehha.ca.gov/chemicals (Accessed, 15 November 2021)

[CR52] Olisah C, Okoh OO, Okoh AI (2019). Global evolution of organochlorine pesticides research in biological and environmental matrices from 1992 to 2018: A bibliometric approach. Emerging Contaminants.

[CR53] Ouyang X, Mao X, Sun C, Du K (2019). Industrial energy efficiency and driving forces behind efficiency improvement: Evidence from the Pearl River Delta urban agglomeration in China. J Clean Prod.

[CR54] Pereira MB, Facco JF, Zemolin GM, Martins ML, Prestes OD, Zanella R, Adaime MB (2014) Pesticide multiresidue determination in rice paddy water by gas chromatography coupled with triple quadrupole mass spectrometry. J AOAC Int 97(4). 10.5740/jaoacint.sgepereira 10.5740/jaoacint.sgepereira 25145127

[CR55] PPDB: the pesticides properties data base (2021) https://sitem.herts.ac.uk/aeru/ppdb/en/ (Accessed, 10 March 2021)

[CR56] Salam MA, Dayal SR, Siddiqua SA, Muhib MI, Bhowmik S, Kabir MM, Rak AAE, Srzednicki G (2021) Risk assessment of heavy metals in marine fish and seafood from Kedah and Selangor coastal regions of Malaysia: a high-risk health concern for consumers. Environ Sci Pollut Res 39:55166–55175. 10.1007/s11356-021-14701-z 10.1007/s11356-021-14701-z 34129166

[CR57] Sapugahawatte DN, Li C, Dharmaratne P, Zhu C, Yeoh YK, Yang J, Lo NWS, Wong KT, Ip M (2022). Prevalence and Characteristics of Streptococcus agalactiae from Freshwater Fish and Pork in Hong Kong Wet Markets. Antibiotics.

[CR58] Smedes F, Thomasen TK (1996) Evaluation of the Bligh and Dyer lipid determination method. Mar Pollut Bull 32:681–688

[CR59] Suárez P, Ruiz Y, Alonso A, San Juan F (2012). Organochlorine compounds in mussels cultured in the Ría of Vigo: Accumulation and origin. Chemosphere.

[CR60] Sun YX, Zhang ZW, Xu XR, Hu YX, Luo XJ, Cai MG, Mai BX (2015). Bioaccumulation and biomagnification of halogenated organic pollutants in mangrove biota from the Pearl River Estuary. South China Marine Pollution Bulletin.

[CR61] Tang D, Liu X, He H, Cui Z, Gan H, Xia Z (2020). Distribution, sources and ecological risks of organochlorine compounds (DDTs, HCHs and PCBs) in surface sediments from the Pearl River Estuary. China Marine Pollution Bulletin.

[CR62] Tang J, An T, Li G, Wei C (2018). Spatial distributions, source apportionment and ecological risk of SVOCs in water and sediment from Xijiang River, Pearl River Delta. Environ Geochem Health.

[CR63] Tian L, Li J, Zhao S, Tang J, Li J, Guo H, Liu X, Zhong G, Xu Y, Lin T, Lyv X (2021). DDT, Chlordane, and Hexachlorobenzene in the air of the pearl river delta revisited: a tale of source, history, and monsoon. Environ Sci Technol.

[CR64] Tsygankov VY (2019). Organochlorine pesticides in marine ecosystems of the Far Eastern Seas of Russia (2000–2017). Water Res.

[CR65] United Nations Environmental Programme (2016) Stockholm convention on persistent organic pollutants. Persistent Organic Pollutants Review Committee. UNEP/POPS/POPRC.12/2

[CR66] US Environmental Protection Agency (EPA) (1998) Guidelines for Ecological Risk Assessment. Washington, 1998

[CR67] Van der Oost R, Beyer J, Vermeulen NPE (2003). Fish bioaccumulation and biomarkers in environmental risk assessment: a review. Environ Toxicol Pharmacol.

[CR68] Wang C, Lu Y, Sun B, Zhang M, Wang C, Xiu C, Johnson AC, Wang P (2022). Ecological and human health risks of antibiotics in marine species through mass transfer from sea to land in a coastal area: A case study in Qinzhou Bay, the South China sea. Environmental Pollution.

[CR69] Wang Z, Zhai F, Wang HJ, Zhang JG, Du WW, Su C, Zhang J, Jiang HR, Zhang B (2015). Secular trends in meat and seafood consumption patterns among Chinese adults, 1991–2011. European Jounal of Clinical Nutricion.

[CR70] Wei S, Lau RKF, Fung CN, Zheng GJ, Lam JCW, Connell DW, Fang Z, Richardson BJ, Lam PKS (2006). Trace organic contamination in biota collected from the Pearl River Estuary, China: A preliminary risk assessment. Mar Pollut Bull.

[CR71] Wong MH, Leung AOW, Chan JKY, Choi MPK (2005) A review on the usage of POP pesticides in China, with emphasis on DDT loadings in human milk. Chemosphere, 60(6), 0–752. 10.1016/j.chemosphere.2005.04.028 10.1016/j.chemosphere.2005.04.028 15949838

[CR72] World Wildlife Fund (n.d.) Sustainable seafood. https://www.wwf.org.hk/en/oceans/seafood/. Accessed 26 Oct 2022

[CR73] Xiao J, Xu X, Wang F, Ma J, Liao M, Shi Y, Fang Q, Cao H (2018). Analysis of exposure to pesticide residues from Traditional Chinese Medicine. J Hazard Mater.

[CR74] Xu L, Granger C, Dong H, Mao Y, Duan S, Li J, Zhimin Z (2020). Occurrences of 29 pesticides in the Huangpu River, China: Highest ecological risk identified in Shanghai metropolitan area. Chemosphere.

[CR75] Xu M, Huang H, Li N, Li F, Wang D, Luo Q (2019). Occurrence and ecological risk of pharmaceuticals and personal care products (PPCPs) and pesticides in typical surface watersheds, China. Ecotoxicol Environ Saf.

[CR76] Yang N, Matsuda M, Kawano M, Wakimoto T (2006). PCBs and organochlorine pesticides (OCPs) in edible fish and shellfish from China. Chemosphere.

[CR77] Yang L, Huang JW, Xue C, Zhou FC (2020) Southwest securities: Hunan Haili Chemical Co., Ltd. (600731) third quarter report. The company’s performance exceeded expectations-2020-10-27. https://pdf.dfcfw.com/pdf/H3_AP202010281424203856_1.pdf?1603877033000.pdf. Accessed 29 Sept 2021

[CR78] Yang Y, Yun X, Liu M, Jiang Y, Li QX, Wang J (2014). Concentrations, distributions, sources, and risk assessment of organochlorine pesticides in surface water of the East Lake, China. Environ Sci Pollut Res.

[CR79] Yao S, Huang J, Zhou H, Cao C, Ai T, Xing H, Sun J (2022). Levels, Distribution and Health Risk Assessment of Organochlorine Pesticides in Agricultural Soils from the Pearl River Delta of China. Int J Environ Res Public Health.

[CR80] Yi X, Zhang C, Liu H, Wu R, Tian D, Ruan J, Zhang T, Huang M, Ying G (2019). Occurrence and distribution of neonicotinoid insecticides in surface water and sediment of the Guangzhou section of the Pearl River, South China. Environ Pollut.

[CR81] Zhang G, Parker A, House A, Mai B, Li X, Kang Y, Wang Z (2002). Sedimentary records of DDT and HCH in the Pearl River Delta, south China. Environ Sci Technol.

[CR82] Zhang J, Liu F, Chen R, Feng T, Dong S, Shen H (2012). Levels of polychlorinated biphenyls and organochlorine pesticides in edible shellfish from Xiamen (China) and estimation of human dietary intake. Food Chem Toxicol.

[CR83] Zheng S, Chen B, Qiu X, Chen M, Ma Z, Yu X (2016). Distribution and risk assessment of 82 pesticides in Jiulong River and estuary in South China. Chemosphere.

