# Supplementary material for: Quantification of insecticides in commercial seafood sold in East Asian markets: risk assessment for consumers
Source: Environ Sci Pollut Res Int. 2022 Dec 14;30(12):34585–97. doi: 10.1007/s11356-022-24413-7 (PMC10017608; doi:10.1007/s11356-022-24413-7)
Supplement: Supplementary file 1 — Supplementary file1 (DOCX 4298 KB) [file 11356_2022_24413_MOESM1_ESM.docx]

**Supplementary material**

**Quantification of insecticides in commercial seafood sold in East Asian markets: risk assessment for consumers**

Lucia Ivorra^a^, Patricia G. Cardoso^b^, Shek Kiu Chan^a^, Catarina Cruzeiro^c,d^*^,1^, Karen Tagulao^a,1^

^a^Institute of Science and Environment, ISE—University of Saint Joseph, Macao SAR

^b^CIIMAR/CIMAR—Interdisciplinary Centre for Marine and Environmental Research, University of Porto, Matosinhos, Portugal

^c^Department of Life Sciences, CFE—Centre for Functional Ecology, University of Coimbra, Coimbra, Portugal

^d^Helmholtz Zentrum München, German Research Centre for Environmental Health, GmbH, Research Unit Comparative Microbiome Analysis, Ingolstaedter Landstrasse 1, 85764, Neuherberg, Germany

*Corresponding author

^1^Both authors contributed equally to this work; joint senior authors

L. Ivorra: 201600297@usj.edu.mo; P.G. Cardoso: pteixeira@ciimar.up.pt; S. Kiu: [skchan@usj.edu.mo](mailto:skchan@usj.edu.mo); C. Cruzeiro: catarina.cruzeiro@helmholtz-muenchen.de; [catarinarcruzeiro@hotmail.com](mailto:catarinarcruzeiro@hotmail.com); K. Tagulao: [karentagulao@usj.edu.mo](mailto:karentagulao@usj.edu.mo)

**1. Standards and reagents.**

LC/GC grade solvents such as methanol (CH_3_OH), acetonitrile (CH_3_CN), ethyl acetate (C_4_H_8_O_2_), chloroform (CHCl_3_) and dichloromethane (CH_2_Cl_2_) were purchased from Merck Limited Company (Germany). Ultrapure water was obtained from a Milli-Q water system (resistance 1= 5.1 μU/cm at 25°C).

**QuEChERS reagents:** anhydrous magnesium sulphate (MgSO_4_), sodium acetate (C_2_H_3_NaO_2_), and Supelclean PSA SPE Bulk Packing (polymerically bonded, ethylenediamine-N-propyl phase that contains both primary and secondary amines), were acquired from Sigma-Aldrich (Steinheim, Germany); MgSO_4_ was preheated (5 h at 500°C) to eliminate residual water and phthalates, as described in Lehotay (2007).

**Pesticide standards:** seafood samples were analysed for 21 insecticides [fenobucarb, pyrimethanil, pirimicarb, aldrin, heptachlor, endosulfan (α- and β-isomer), dieldrin, endrin, methoxychlor, 4,4’-dichlorodiphenyltrichloroethane (4,4’-DDT) and hexachlorocyclohexane (α-, β- and γ-isomer)] and related sub-products, such as endrin ketone, endrin aldehyde, endosulfan sulfate, heptachlor epoxide, dichlorodiphenyldichloroethylene (4,4’-DDE), dichlorodiphenyldichloroethane (4,4’-DDD), and 4,4’-dichlorobenzophenone (4,4’-DCBP). Pirimicarb-*d_6_* and dicofol-*d_8_* were used as internal standard (IS) at a final concentration of 5 μg/L in the matrix; their molecular structure allowed covering all the target compounds, and retention time intervals, 8-13.49 min for pirimicarb-*d_6_* and 13.50-23 min for dicofol-*d_8_.* All standard and IS solutions (98-99%) were acquired from Sigma-Aldrich (Seelze, Germany). Stock solutions were kept in dark at -20°C and were individually prepared in CH_3_OH with 0.1% acetic acid (CH_3_COOH; Sigma-Aldrich, USA) reaching a final concentration of 1000 μg/L.

**Protectants:** D-sorbitol and 3-ethoxy-1,2-propanediol (used as protectants) were purchased from Sigma-Aldrich (Steinheim, Germany); stock solutions of 182 mg/mL in 70% CH_3_OH:H_2_0 and 800 000 mg/L in 100% CH_3_OH were prepared, respectively. Protectants were used as 0.1:1 mg/mL (D-sorbitol:3-ethoxy-1,2-propanediol). 3-ethoxy-1,2-propanediol were kept at 4°C, and D-sorbitol and the protectant mixture were stored in the dark at -20°C.

For quantification purposes, an aliquot of each extracted sample (195 μL) was taken and mixed with a protectants' solution (5 μL) at a final concentration of 0.0025:0.025 g/mL, respectively.

**2. Market’s detail acquisition**

**Fig. S1** Map of the Pearl River Delta region and the distribution of the local markets in Macao (A to C) and Hong Kong (A to D). Adapted from QGIS 2.18 Desktop, version 2.18.15.

**
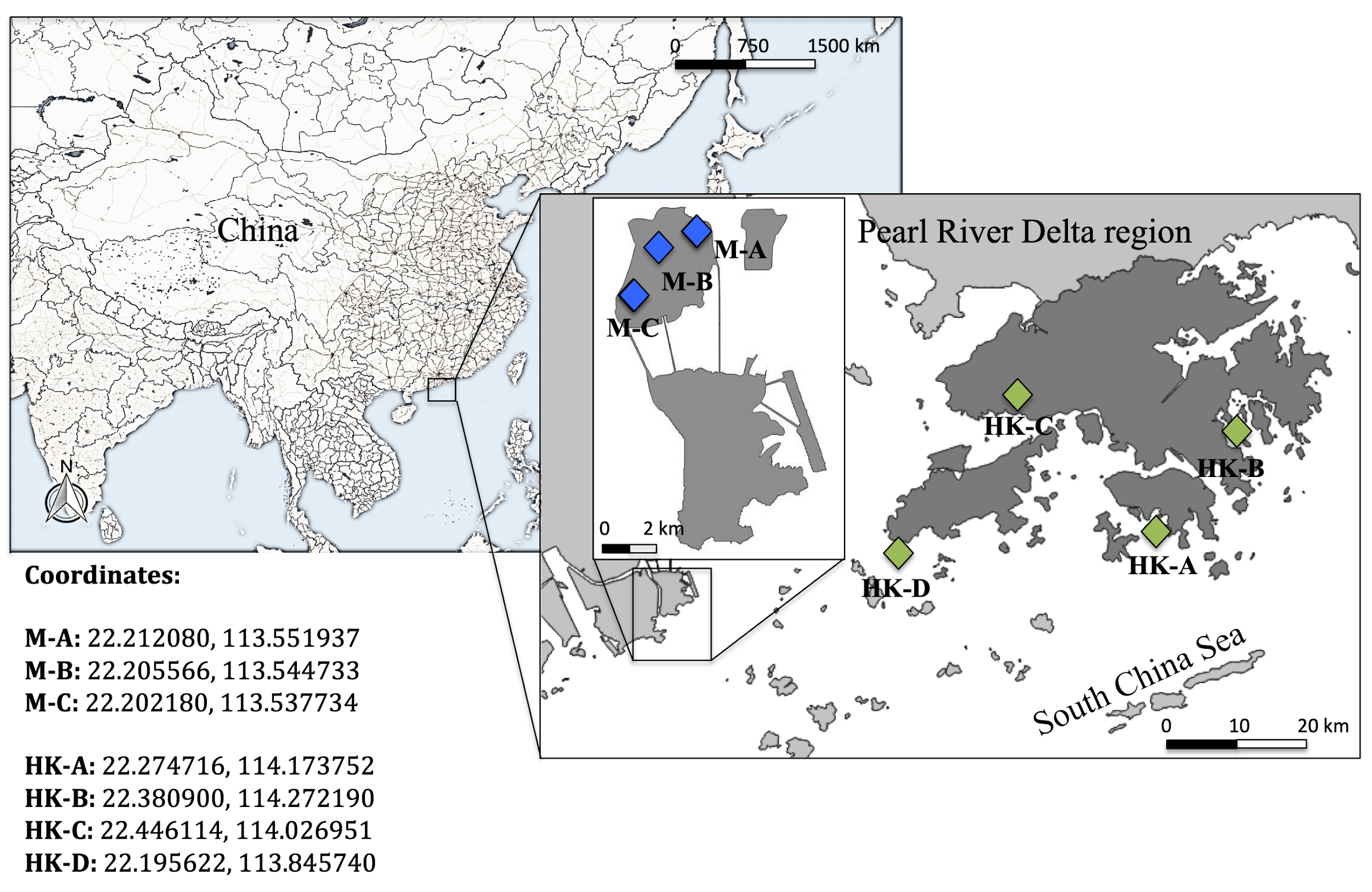
**

**Table S1.** Information regarding sample collection in local seafood markets from Macao and Hong Kong. Numbers indicate the number of suppliers, and letters correspond to the market code for each location.

|  |  |  | **Macao** | | | **Hong Kong** | | | |
| --- | --- | --- | --- | --- | --- | --- | --- | --- | --- |
| **Category** |  |  | **A** | **B** | **C** | **A** | **B** | **C** | **D** |
| Bivalves | |  |  |  |  |  |  |  |  |
|  | *Meretrix meretrix* (Clams) | | 2 | 2 | 2 | 2 | 1 | 2 | 1 |
|  | *Anadara subcrenata* (Coockles) | | 2 | 2 | 1 | - | - | - | - |
|  | *Perna viridis* (Mussels) | | - | 1 | 2 | 2 | 2 | 2 | 2 |
| Crustaceans |  |  |  |  |  |  |  |  |  |
|  | *Scylla serrate* (Mud crab) | | 2 | 2 | 2 | 2 | 2 | 2 | 1 |
|  | *Portunus pelagicus* (Blue crab) | | 2 | 2 | 2 | 2 | 2 | 2 | 1 |
|  | *Metapenaeus ensis* (Shrimp) | | 2 | 2 | 2 | 2 | 2 | 2 | 1 |
| Fish |  |  |  |  |  |  |  |  |  |
|  | *Boleophthalmus pectinirostris*  (Mudskipper) | | 1 | - | 1 | - | - | - | - |

**Table S2.** Information about the molecular weight, retention time (regarding the bivalves matrix) and ions from the different target compounds. Product ions were used for characterization and quantification purposes. The compound in bold refers to the internal standard used during validation and posterior analysis.

| **Target compound** | **Molecular mass** | **RT** | **GC-MS/MS** | | **CE** | **Ranges** |
| --- | --- | --- | --- | --- | --- | --- |
|  | **(g/mol)** | **(min)** | **Precursor** | **Product** |  |  |
| Fenobucarb | 207.3 | 9.42 | 121.1 | 77.1 | 20.0 | 6.0 |
| α-HCH | 290.8 | 10.55 | 181.0 | 145.0 | 15.0 |  |
| β-HCH | 290.8 | 11.07 | 218.9 | 183.0 | 10.0 |  |
| γ-HCH | 290.8 | 11.28 | 183.0 | 147.0 | 10.0 |  |
| Pyrimethanil | 199.1 | 11.70 | 199.2 | 198.2 | 10.0 | 11.5 |
| **Pirimicarb-D6** | **244.3** | **12.04** | **166.1** | **96.1** | **10.0** |  |
| Pirimicarb | 238.4 | 12.08 | 238.2 | 166.2 | 10.0 |  |
| Heptachlor | 373.3 | 13.18 | 100.0 | 65.1 | 10.0 |  |
| Aldrin | 364.9 | 14.21 | 262.8 | 192.9 | 30.0 | 14.0 |
| **Dicofol-D8** | **378.5** | **14.42** | **143.0** | **115.0** | **15.0** |  |
| 4,4’-DCBP | 252.1 | 14.50 | 141.0 | 113.0 | 15.0 |  |
| Heptachlor epoxide | 389.3 | 15.44 | 182.9 | 155.0 | 15.0 |  |
| α-endosulfan | 406.9 | 16.65 | 240.9 | 206.0 | 10.0 | 16.0 |
| 4,4’-DDD | 320.0 | 17.03 | 165.1 | 164.1 | 20.0 |  |
| 4,4’-DDE | 318.0 | 17.15 | 246.0 | 176.1 | 25.0 |  |
| Dieldrin | 380.9 | 17.35 | 262.9 | 193.0 | 30.0 |  |
| Endrin | 380.9 | 18.03 | 262.9 | 193.0 | 30.0 | 18.0 |
| β-endosulfan | 406.9 | 18.37 | 195.0 | 159.0 | 10.0 |  |
| 4,4’-DDT | 320.0 | 18.52 | 235.0 | 165.1 | 30.0 |  |
| Endosulfan sulfate | 422.9 | 19.70 | 271.8 | 236.9 | 10.0 |  |
| Methoxychlor | 345.7 | 20.25 | 227.1 | 169.1 | 25.0 |  |
|  |  |  |  |  |  |  |

**Fig. S2** Chromatograms (I and II) of the spiked bivalve matrix and a commercial one (mussels from Hong Kong), respectively; the number in the full scan means 1- fenobucarb, 2- β-HCH, 3- pyrimethanil, 4- pirimicarb, 5- dicofol-d8, 6- 4,4’-DDE, 7- 4,4’-DDT, 8- endosulfan sulfate, 9- methoxychlor.


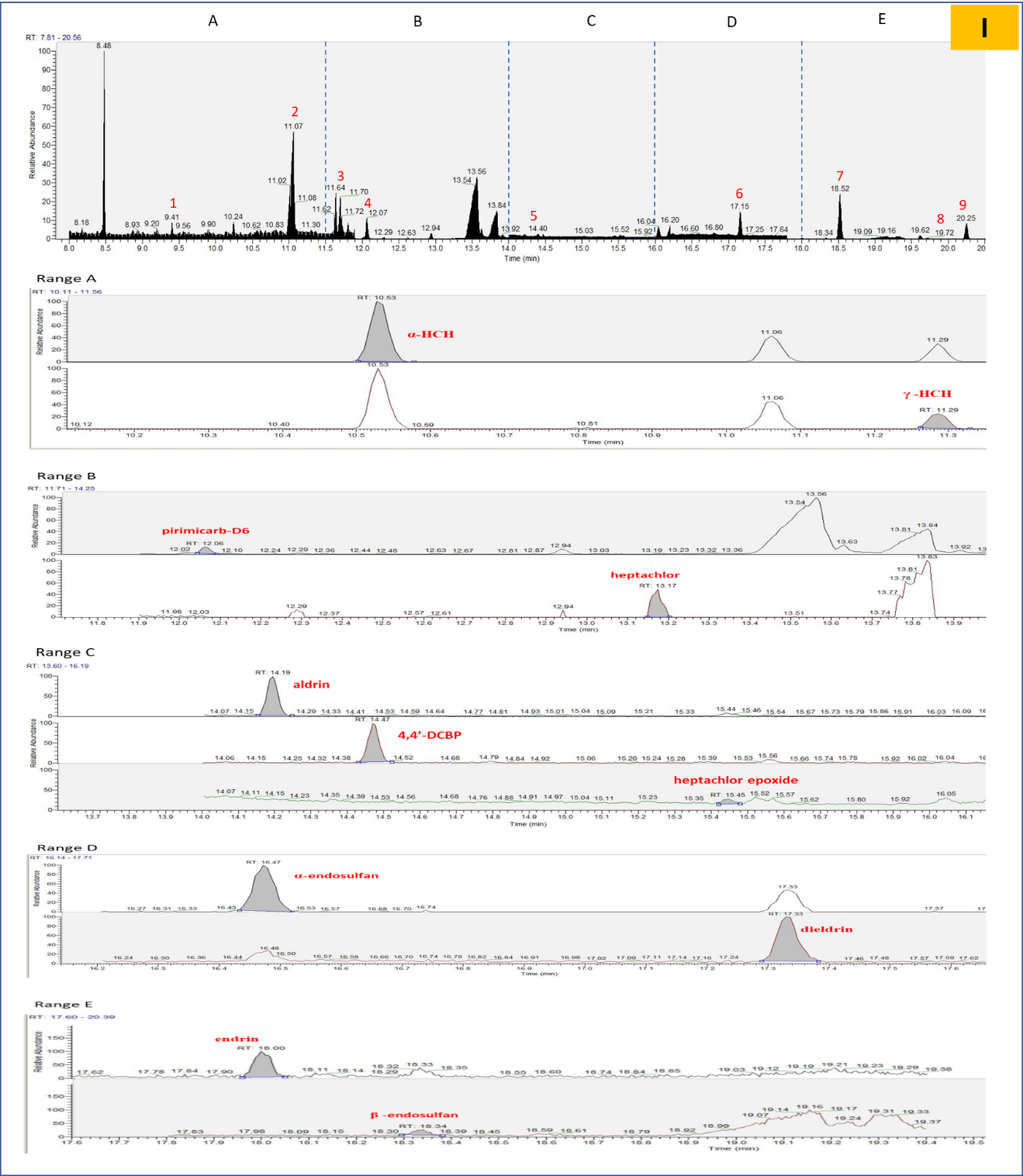


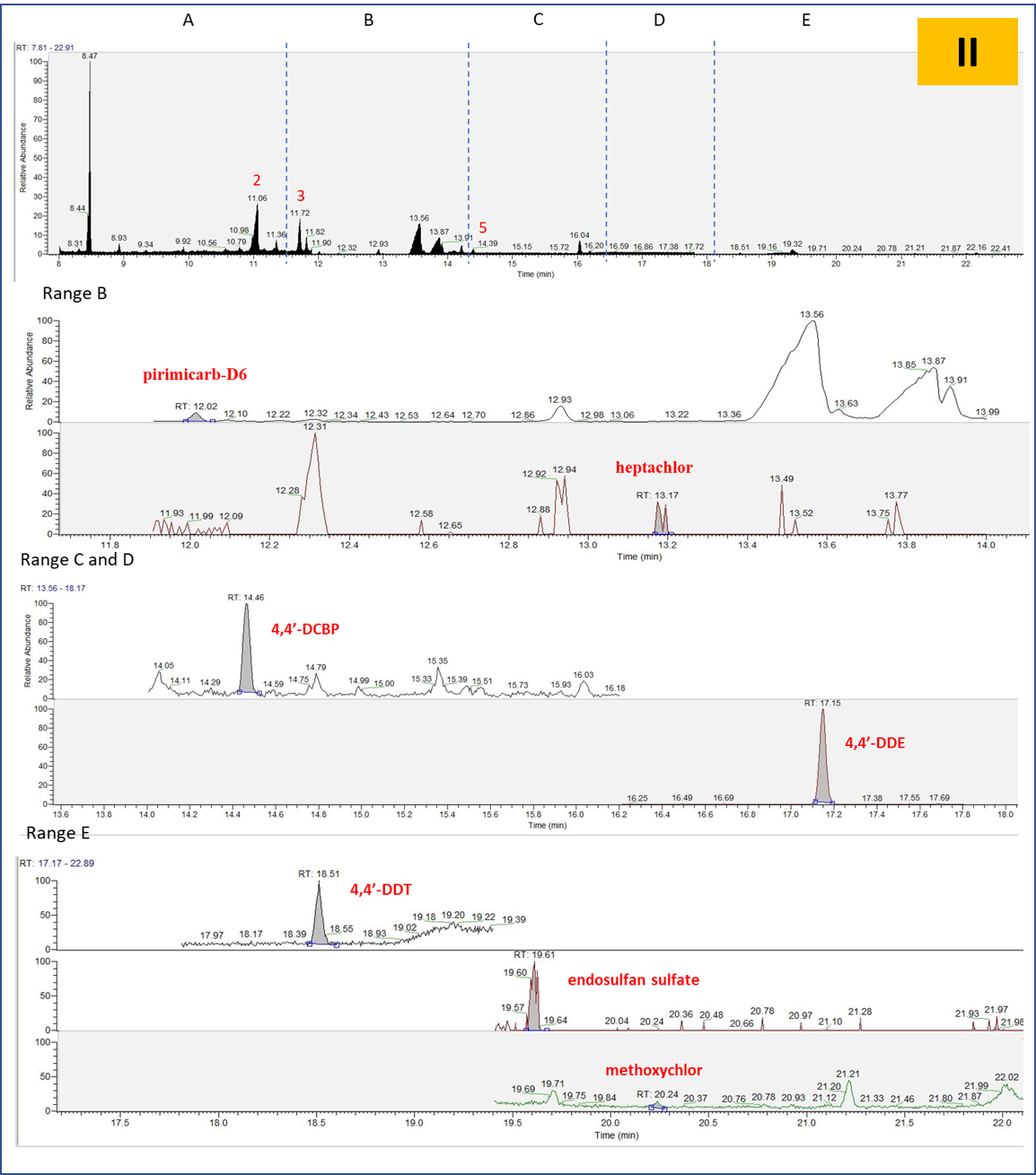


**Table S3.** R^2^ values of the calibration curves for each compound and matrix used for quantification purposes.

| **Compound** | **Bivalves** | **Crabs** | **Shrimps** | **Mudskippers** |
| --- | --- | --- | --- | --- |
| Fenobucarb | 0.993 | 0.918 | 0.933 | 0.875 |
| a-HCH | 0.963 | 0.993 | 0.971 | 0.975 |
| b-HCH | 0.929 | 0.969 | 0.956 | 0.936 |
| Lindane | - | 0.996 | 0.937 | 0.979 |
| Pyrimethanil | 0.999 | 0.930 | 0.983 | 0.974 |
| Pirimircarb | 0.999 | 0.987 | 0.993 | 0.995 |
| Heptachlor | - | 0.996 | 0.988 | 0.979 |
| Aldrin | 0.883 | 0.980 | 0.985 | 0.974 |
| 4,4'-DCBP | 0.986 | 0.856 | 0.981 | 0.847 |
| Heptachlor epoxide | 0.962 | 0.909 | 0.987 | 0.848 |
| a-endosulfan | 0.927 | 0.977 | 0.992 | 0.954 |
| 4,4'-DDD | - | - | - | - |
| 4,4'-DDE | 0.886 | 0.911 | 0.977 | 0.943 |
| Dieldrin | 0.881 | 0.990 | 0.988 | 0.992 |
| Endrin ketone | - | - | - | - |
| Endrin | 0.904 | 0.992 | 0.979 | 0.981 |
| b-endosulfan | 0.953 | 0.96 | 0.97 | 0.917 |
| Endrin aldehyde | - | - | - | - |
| Endosulfan sulfate | 0.980 | 0.876 | 0.956 | 0.966 |
| 4,4'-DDT | 0.937 | 0.926 | 0.958 | 0.967 |
| Methoxychlor | 0.908 | 0.985 | 0.943 | 0.920 |

**Table S4.** Results of LOD, LOQ and QC for the different matrices used during validation. Value regarding QC was established as 5 times LOQ.

| **Compound** | **RT** | **Bivalves (ng/g WW)** | | | **Crab (ng/g WW)** | | | **Shrimp (ng/g WW)** | | | **Fish (ng/g WW)** | | |
| --- | --- | --- | --- | --- | --- | --- | --- | --- | --- | --- | --- | --- | --- |
|  |  | **LOD** | **LOQ** | **QC** | **LOD** | **LOQ** | **QC** | **LOD** | **LOQ** | **QC** | **LOD** | **LOQ** | **QC** |
| Fenobucarb | 9.39 | 0.62 | 1.89 | **9.45** | 0.17 | 0.53 | **2.63** | 0.54 | 1.64 | **8.19** | 0.38 | 1.14 | **5.69** |
| α-HCH | 10.53 | 0.78 | 2.38 | **14.00** | 0.19 | 0.58 | **2.90** | 0.23 | 0.71 | **3.55** | 0.25 | 0.77 | **3.85** |
| β-HCH | 11.06 | 0.08 | 0.24 | **1.20** | 0.09 | 0.27 | **1.35** | 0.51 | 1.54 | **7.71** | 0.79 | 2.40 | **11.99** |
| γ-HCH | 11.29 | 0.2 | 0.61 | **3.05** | 0.12 | 0.36 | **1.81** | 0.29 | 0.88 | **4.41** | 0.31 | 0.95 | **4.76** |
| Pyrimethanil | 11.61 | 0.57 | 1.73 | **8.65** | 0.30 | 0.90 | **4.52** | 0.09 | 0.26 | **1.30** | 0.75 | 2.28 | **11.41** |
| Pirimicarb | 12.06 | 0.06 | 0.17 | **0.85** | 0.17 | 0.51 | **2.54** | 0.06 | 0.17 | **0.86** | 0.14 | 0.43 | **2.13** |
| Heptachlor | 13.16 | 1.41 | 4.27 | **21.35** | 0.06 | 0.18 | **0.89** | 0.11 | 0.34 | **1.68** | 0.24 | 0.74 | **3.68** |
| Aldrin | 14.20 | 0.63 | 1.91 | **9.55** | 0.26 | 0.78 | **3.92** | 0.33 | 0.99 | **4.94** | 0.35 | 1.07 | **5.35** |
| 4,4’-DCBP | 14.50 | 1.33 | 4.02 | **20.10** | 0.39 | 1.18 | **5.90** | 0.62 | 1.89 | **9.44** | 0.63 | 1.91 | **9.54** |
| Heptachlor epoxide | 15.43 | 0.23 | 0.69 | **3.45** | 0.11 | 0.33 | **1.65** | 0.61 | 1.86 | **9.31** | 0.45 | 1.37 | **6.83** |
| α -endosulfan | 16.48 | 0.91 | 2.76 | **13.08** | 0.07 | 0.22 | **1.09** | 0.35 | 1.06 | **5.32** | 0.62 | 1.87 | **9.35** |
| 4,4’-DDE + 4,4’-DDD | 17.17 | 0.78 | 2.36 | **11.80** | 0.81 | 2.46 | **12.32** | 0.39 | 1.19 | **5.95** | 1.49 | 4.51 | **22.56** |
| Dieldrin | 17.32 | 0.54 | 1.63 | **8.10** | 0.01 | 0.03 | **0.14** | 0.40 | 1.21 | **6.04** | 0.12 | 0.35 | **1.75** |
| Endrin | 18.02 | 1.34 | 4.05 | **20.25** | 0.11 | 0.34 | **1.70** | 0.49 | 1.50 | **7.49** | 0.26 | 0.78 | **3.91** |
| β-endosulfan | 18.34 | 0.33 | 1.00 | **5.00** | 0.20 | 0.61 | **3.03** | 0.36 | 1.10 | **5.48** | 0.06 | 0.18 | **0.88** |
| Ensodulfan sulfate | 19.64 | 1.63 | 4.95 | **24.75** | 0.20 | 0.61 | **3.03** | 0.43 | 1.31 | **6.55** | 0.68 | 2.06 | **10.28** |
| 4,4’-DDT | 18.52 | 0.20 | 0.60 | **3.10** | 0.18 | 0.55 | **2.77** | 0.29 | 0.89 | **4.46** | 0.38 | 1.15 | **5.74** |
| Methoxychlor | 20.26 | 0.31 | 0.94 | **4.50** | 0.81 | 2.46 | **12.31** | 0.96 | 2.91 | **14.57** | 0.33 | 1.01 | **5.06** |

**Table S5.** Results of Recovery, RSD and Accuracy for bivalves’ matrix used during validation considering two QCs (2xLOQ and 4xLOQ).

| **Target compounds** | **QCs** | | **Recovery** | **SD** | **RSD** | **SD** | **Accuracy** | **SD** |
| --- | --- | --- | --- | --- | --- | --- | --- | --- |
|  | **(ng/g WW)** | | **(%)** |  | **(%)** |  | **(%)** |  |
| α- HCH | 2xLOQ | 4.76 | 67.02 | 20.52 | 7.75 | 7.71 | 79.64 | 19.26 |
|  | 4xLOQ | 9.51 | 99.17 | 14.33 | 5.67 | 3.61 | 113.11 | 15.79 |
| Fenobucarb | 2xLOQ | 1.89 | 99.44 | 14.38 | 5.16 | 1.54 | 80.47 | 5.53 |
|  | 4xLOQ | 7.55 | 95.83 | 8.65 | 2.64 | 0.83 | 111.79 | 8.11 |
| β-HCH | 2xLOQ | 0.24 | 86.17 | 15.93 | 4.31 | 2.35 | 106.22 | 10.77 |
|  | 4xLOQ | 0.96 | 87.45 | 13.33 | 2.78 | 0.53 | 78.65 | 10.22 |
| γ-HCH | 2xLOQ | 0.61 | 91.83 | 4.96 | 13.65 | 0.51 | 80.68 | 17.42 |
|  | 4xLOQ | 2.44 | 113.25 | 5.01 | 8.22 | 1.97 | 64.13 | 2.43 |
| Pyrimethanil | 2xLOQ | 1.73 | 98.31 | 12.15 | 6.45 | 2.92 | 69.11 | 5.44 |
|  | 4xLOQ | 6.92 | 70.09 | 12.20 | 1.56 | 0.71 | 110.51 | 4.18 |
| Pirimicarb | 2xLOQ | 0.17 | 109.73 | 18.96 | 8.97 | 2.56 | 84.05 | 5.61 |
|  | 4xLOQ | 0.68 | 73.25 | 13.28 | 3.28 | 0.88 | 84.76 | 4.35 |
| Heptachlor | 2xLOQ | 8.53 | 83.39 | 13.67 | 8.72 | 7.16 | 111.29 | 33.73 |
|  | 4xLOQ | 17.07 | 85.91 | 18.57 | 9.23 | 1.71 | 90.11 | 18.86 |
| Aldrin | 2xLOQ | 1.91 | 97.79 | 12.88 | 17.56 | 13.27 | 82.45 | 15.16 |
|  | 4xLOQ | 7.64 | 103.42 | 12.35 | 4.07 | 0.69 | 103.18 | 24.83 |
| 4,4’-DCBP | 2xLOQ | 4.02 | 99.02 | 20.56 | 4.85 | 1.60 | 87.84 | 17.91 |
|  | 4xLOQ | 16.08 | 117.73 | 17.17 | 3.53 | 1.49 | 74.67 | 10.22 |
| Heptachlor epoxide | 2xLOQ | 0.69 | 94.67 | 30.78 | 16.38 | 1.76 | 78.22 | 10.02 |
|  | 4xLOQ | 2.76 | 103.19 | 16.80 | 12.23 | 5.14 | 72.95 | 9.63 |
| α-endosulfan | 2xLOQ | 5.52 | 112.73 | 18.37 | 5.18 | 5.32 | 73.74 | 17.41 |
|  | 4xLOQ | 11.03 | 111.14 | 4.42 | 3.86 | 0.90 | 65.02 | 4.67 |
| 4,4’-DDE + 4,4’-DDD | 2xLOQ | 4.72 | 87.14 | 8.48 | 5.26 | 3.01 | 84.44 | 10.75 |
|  | 4xLOQ | 9.43 | 110.17 | 5.16 | 4.30 | 3.95 | 61.65 | 3.33 |
| Dieldrin | 2xLOQ | 1.63 | 104.78 | 2.27 | 10.98 | 4.53 | 86.09 | 12.65 |
|  | 4xLOQ | 6.52 | 97.01 | 19.52 | 4.40 | 1.36 | 75.86 | 7.81 |
| Endrin | 2xLOQ | 8.10 | 103.45 | 13.33 | 7.16 | 3.29 | 96.38 | 13.32 |
|  | 4xLOQ | 16.20 | 78.29 | 6.91 | 5.54 | 2.99 | 104.03 | 9.48 |
| β-endosulfan | 2xLOQ | 1.00 | 80.76 | 13.66 | 13.06 | 1.87 | 120.62 | 23.32 |
|  | 4xLOQ | 4.00 | 91.33 | 26.45 | 8.83 | 1.94 | 91.96 | 19.03 |
| 4,4’-DDT | 2xLOQ | 0.60 | 105.71 | 23.24 | 4.21 | 3.42 | 81.88 | 18.74 |
|  | 4xLOQ | 2.41 | 105.19 | 13.37 | 4.48 | 2.21 | 97.23 | 18.58 |
| Endosulfan sulfate | 2xLOQ | 9.90 | 94.37 | 10.65 | 10.90 | 5.89 | 63.50 | 6.64 |
|  | 4xLOQ | 19.80 | 86.22 | 5.90 | 12.03 | 8.44 | 93.04 | 9.51 |
| Methoxychlor | 2xLOQ | 1.88 | 102.83 | 15.73 | 4.92 | 0.74 | 78.61 | 35.75 |
|  | 4xLOQ | 3.75 | 114.83 | 6.69 | 3.37 | 0.34 | 92.82 | 15.93 |


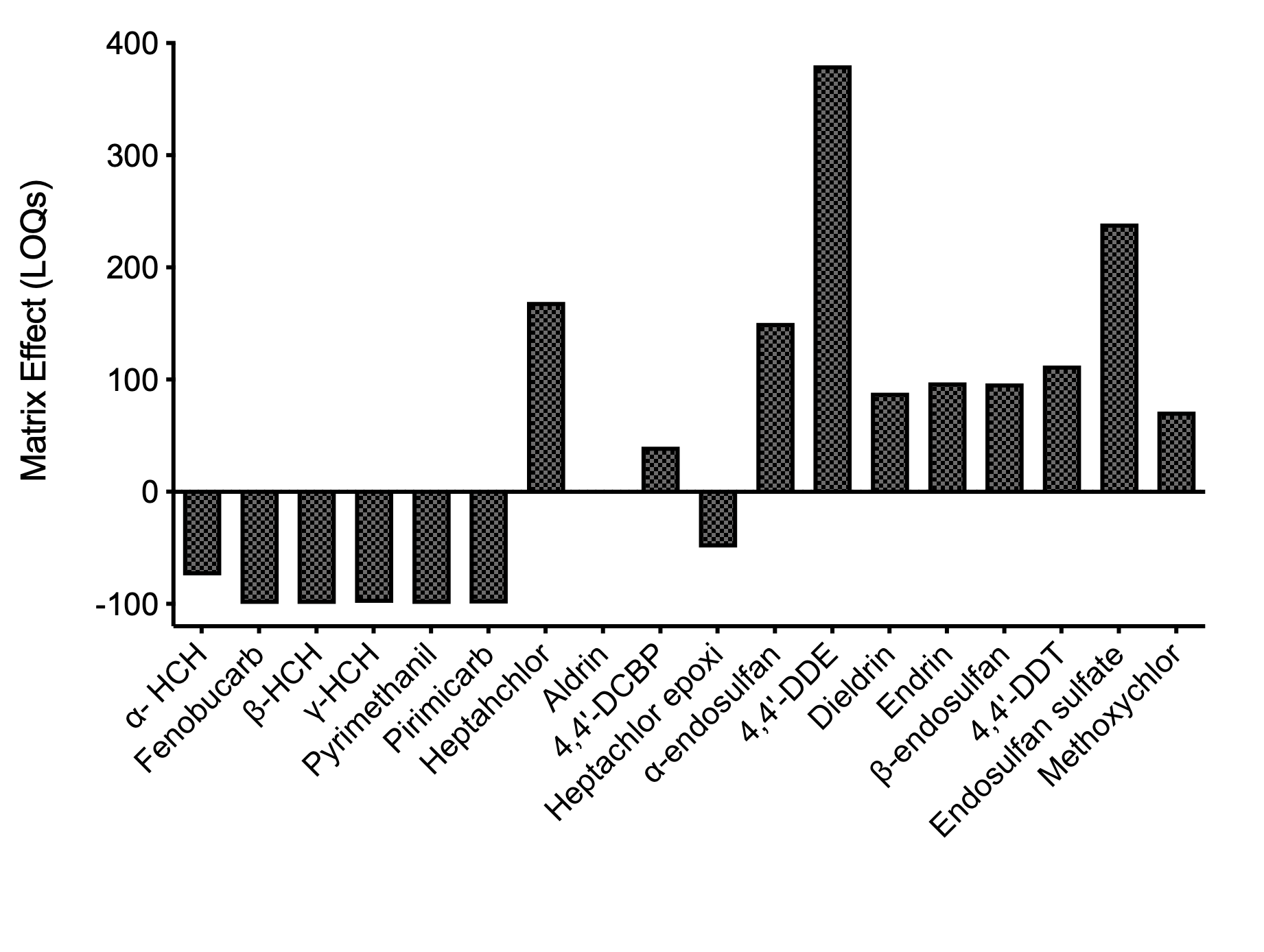


**Fig. S3** Evaluation of Matrix Effect at LOQ concentration for all the OCPs selected; the results are expressed as percentages (%).

**Table S6.** Information regarding the lipid content of the seafood samples collected in Macao and Hon Kong markets. Data are expressed in % of lipid and g/g of WW.

| **Category** | **Species** |  | **Average Lipid content** |  |
| --- | --- | --- | --- | --- |
|  |  | **%** | **(g/g of WW)** | **SD** |
| Bivalves | *Meretrix meretrix* | 5.812 | 0.058 | 0.006 |
|  | *Anadara subcrenata* | 5.480 | 0.055 | 0.008 |
|  | *Perna viridis* | 6.088 | 0.061 | 0.003 |
| Crustaceans | *Portunus pelagicus* | 9.728 | 0.097 | 0.010 |
|  | *Scylla serrata* | 5.212 | 0.052 | 0.005 |
|  | *Metapenaeus ensis* | 4.700 | 0.047 | 0.005 |
| Fish | *Boleophthalmus pectinorostris* | 7.004 | 0.070 | 0.003 |


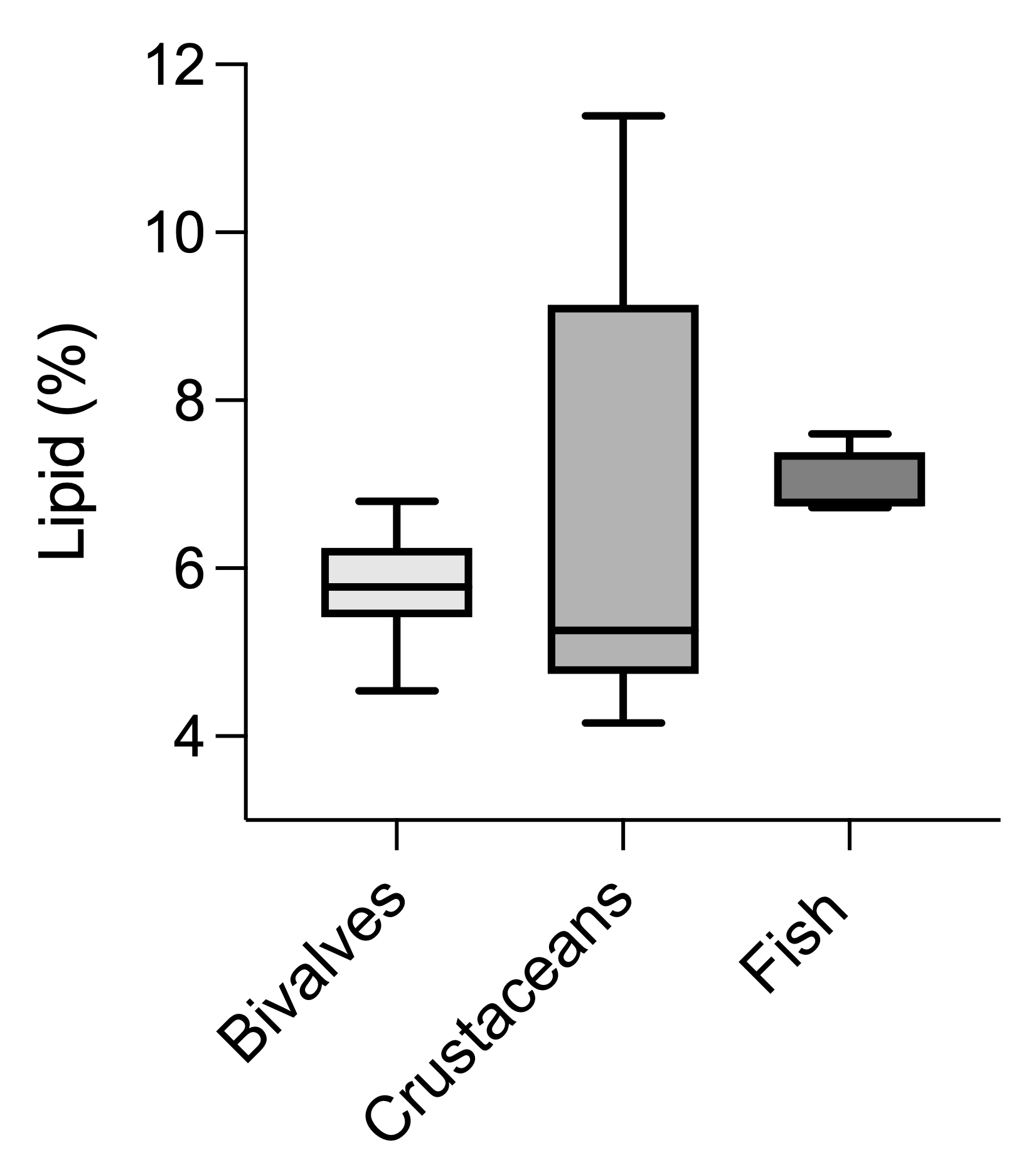


a

a

a

**Fig. S4** Percentage of lipids (%) quantified in bivalves, crustaceans and fish collected from Hong Kong and Macao’s markets.
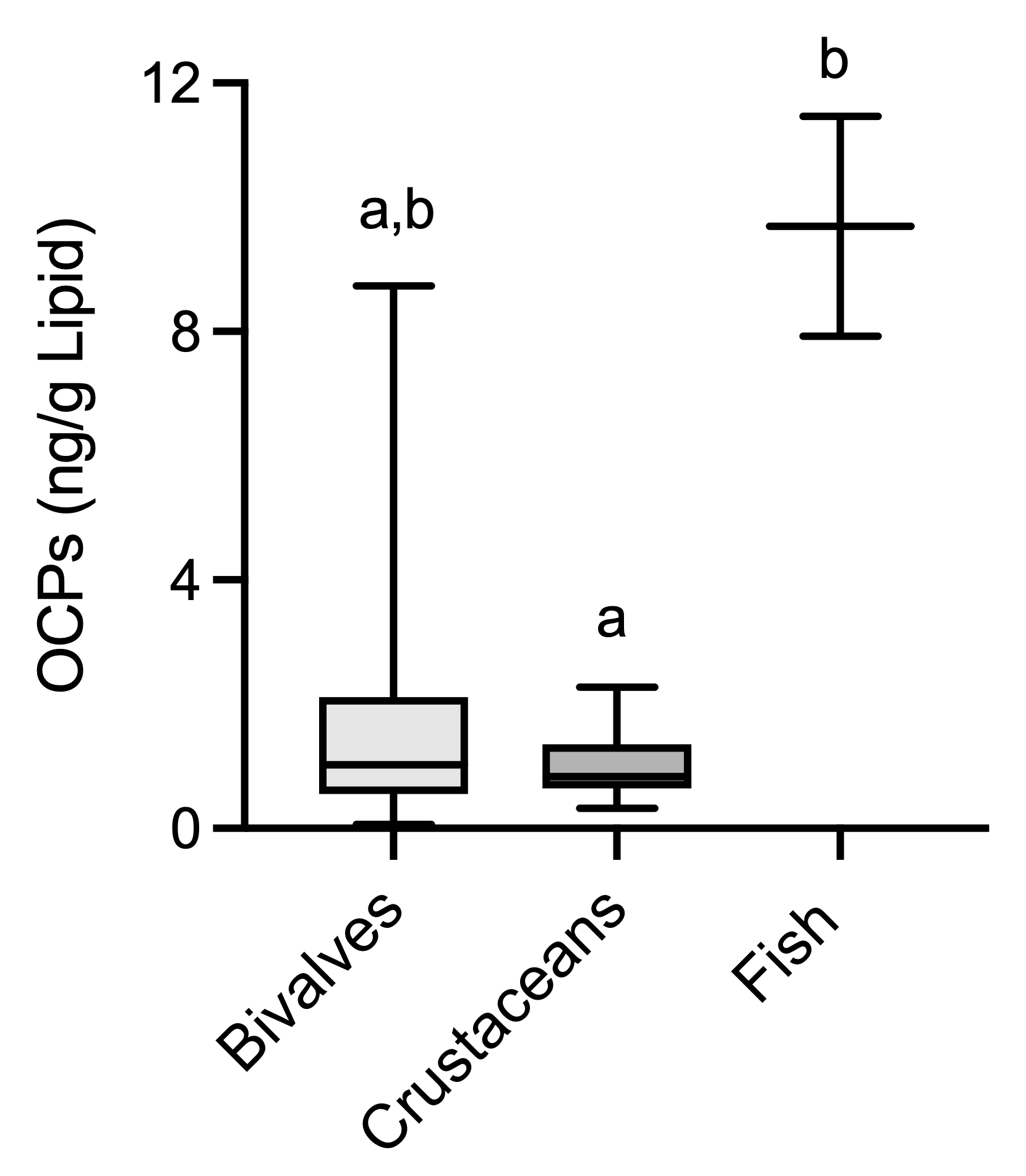


**Fig. S5** Concentration of insecticides (ng/g Lipid) quantified in bivalves, crustaceans and fish collected from Hong Kong and Macao’s markets and normalized by the lipid content of each category. Letters indicated significant differences among categories (Kruskal-Wallis, p<0.05).

**Table S7.** Hazard quotients (HQ), for adults, considering long-term (LT) and short-term (ST) exposure to pollutants, and hazard index (HI) of the OCPs concentrations quantified in fish samples from Hong Kong and Macao markets. EADI: estimated average daily intake; ADI: acceptable daily intake; EMDI: estimated max. daily intake; ArfD: acute reference dose; EC: Environmental concentration; na: not available. (-) Indicates not information available to proceed with the corresponding calculations.

| **Compound** | **HQ-LT (chronic)** | | | | **HQ-ST (acute)** | | | |
| --- | --- | --- | --- | --- | --- | --- | --- | --- |
|  | **Average-EC**  **(mg/kg WW)** | **EADI** | **ADI** | **HQ=EADI/ADI** | **Max-EC**  **(mg/kg WW)** | **EMDI** | **ARfD** | **HQ=EMDI/ARfD** |
| ∑aldrin+dieldrin | na | na | 1.0E-04 | - | na | na | 3.0E-03 | - |
| Fenobucarb | 2.2E-01 | 4.0E-04 | 6.0E-02 | 6.7E-03 | 4.1E-01 | 6.8E-04 | na | - |
| ∑DDD,DDE,DDT | 6.5E-02 | 1.1E-04 | 5.0E-03 | 2.2E-02 | 1.7E-01 | 2.9E-04 | na | - |
| Endosulfan (α+β+sulphate) | na | na | 6.0E-03 | - | na | na | 2.0E-02 | - |
| Methoxychlor | 9.9E-03 | 1.7E-05 | 1.0E-01 | 1.7E-04 | 1.0E-02 | 1.7E-05 | 5.0E-03 | 3.4E-03 |
| Heptachlor | na | na | 1.0E-04 | - | na | na | na | - |
| HCH (α) | na | na | 5.0E-03 | - | na | na | 6.0E-03 | - |
| HCH (β) | 2.1E-02 | 3.5E-05 | 5.0E-03 | 7.0E-03 | 3.1E-02 | 5.2E-05 | 6.0E-03 | 8.7E-03 |
| Lindane | na | na | 8.0E-03 | - | na | na | 3.0E-04 | - |
| Heptachlor epoxide | 9.7E-02 | 1.6E-04 | 1.0E-04 | **1.6E+00** | 1.1E-01 | 1.8E-04 | na | - |
| 4,4'-DCBP | na | na | na | - | na | na | na | - |
| Endrin | na | na | 2.0E-04 | - | na | na | 3.0E-04 | - |
| Pirimicarb | 3.9E-03 | 6.5E-06 | 3.5E-02 | 1.8E-04 | 4.0E-03 | 6.6E-06 | 1.0E-01 | 6.6E-05 |
| Pyrimethanil | na | na | 1.7E-01 | - | na | na | 8.5E-01 | - |
| **HI** |  |  |  | **1.6E+00** |  |  |  |  |

**Table S8.** Hazard quotients (HQ), for adults, considering long-term (LT) and short-term (ST) exposure to pollutants, and hazard index (HI) of the OCPs concentrations quantified in bivalve samples from Hong Kong and Macao markets. EADI: estimated average daily intake; ADI: acceptable daily intake; EMDI: estimated max. daily intake; ArfD: acute reference dose; EC: Environmental concentration; na: not available.

| **Compound** | **HQ-LT (chronic)** | | | | **HQ-ST (acute)** | | | |
| --- | --- | --- | --- | --- | --- | --- | --- | --- |
|  | **Average-EC**  **(mg/kg WW)** | **EADI** | **ADI** | **HQ=EADI/ADI** | **Max-EC**  **(mg/kg WW)** | **EMDI** | **ARfD** | **HQ=EMDI/ARfD** |
| ∑aldrin+dieldrin | 4.5E-03 | 7.5E-06 | 1.0E-04 | 7.5E-02 | 6.8E-03 | 1.1E-05 | 3.0E-03 | 3.8E-03 |
| Fenobucarb | 1.9E-03 | 3.2E-06 | 6.0E-02 | 5.4E-05 | 5.9E-03 | 9.8E-06 | na | - |
| ∑DDD,DDE,DDT | 5.8E-03 | 9.6E-06 | 5.0E-03 | 1.9E-03 | 1.0E-02 | 1.7E-05 | na | - |
| Endosulfan (α+β+sulphate) | na | na | 6.0E-03 | - | na | na | 2.0E-02 | - |
| Methoxychlor | 1.3E-03 | 2.1E-06 | 1.0E-01 | 2.1E-05 | 2.0E-03 | 3.3E-06 | 5.0E-03 | 6.5E-04 |
| Heptachlor | na | na | 1.0E-04 | - | na | na | na | - |
| HCH (α) | na | na | 5.0E-03 |  | na | na | 6.0E-03 | - |
| HCH (β) | 2.8E-02 | 4.7E-05 | 5.0E-03 | 9.4E-03 | 9.5E-02 | 1.6E-04 | 6.0E-03 | 2.6E-02 |
| Lindane | na | na | 8.0E-03 | - | na | na | 3.0E-04 | - |
| Heptachlor epoxide | 5.5E-02 | 9.1E-05 | 1.0E-04 | 9.1E-01 | 2.2E-01 | 3.6E-04 | na | - |
| 4,4'-DCBP | 2.0E-02 | 3.3E-05 | na | - | 4.8E-02 | 7.9E-05 | na | - |
| Endrin | 6.4E-04 | 1.1E-06 | 2.0E-04 | 5.3E-03 | 1.2E-03 | 2.0E-06 | 3.0E-04 | 6.7E-03 |
| Pirimicarb | 4.0E-04 | 6.7E-07 | 3.5E-02 | 1.9E-05 | 4.1E-04 | 6.8E-07 | 1.0E-01 | 6.8E-06 |
| Pyrimethanil | 1.1E-04 | 1.8E-07 | 1.7E-01 | 1.1E-06 | 1.1E-04 | 1.8E-07 | 8.5E-01 | 2.2E-07 |
| **HI** |  |  |  | **1.0E+00** |  |  |  |  |

**Table S9.** Hazard quotients (HQ), for adults, considering long-term (LT) and short-term (ST) exposure to pollutants, and hazard index (HI) of the OCPs concentrations quantified in crustacean samples from Hong Kong and Macao markets. EADI: estimated average daily intake; ADI: acceptable daily intake; EMDI: estimated max. daily intake; ArfD: acute reference dose; EC: Environmental concentration; na: not available.

| **Compound** | **HQ-LT (chronic)** | | | | **HQ-ST (acute)** | | | |
| --- | --- | --- | --- | --- | --- | --- | --- | --- |
|  | **Average-EC**  **(mg/kg WW)** | **EADI** | **ADI** | **HQ=EADI/ADI** | **Max-EC**  **(mg/kg WW)** | **EMDI** | **ARfD** | **HQ=EMDI/ARfD** |
| ∑aldrin+dieldrin | 0.004 | 7.4E-06 | 1.0E-04 | 7.4E-02 | 8.3E-03 | 1.4E-05 | 3.0E-03 | 4.6E-03 |
| Fenobucarb | 0.004 | 6.3E-06 | 6.0E-02 | 1.0E-04 | 4.3E-03 | 7.1E-06 | na | - |
| ∑DDD,DDE,DDT | 0.040 | 6.6E-05 | 5.0E-03 | 1.3E-02 | 1.7E-01 | 2.9E-04 | na | - |
| Endosulfan (α+β+sulphate) | 0.005 | 7.6E-06 | 6.0E-03 | 1.3E-03 | 2.3E-02 | 9.5E-06 | 2.0E-02 | 4.8E-04 |
| Methoxychlor | 0.006 | 1.1E-05 | 1.0E-01 | 1.1E-04 | 6.4E-03 | 1.1E-05 | 5.0E-03 | 2.1E-03 |
| Heptachlor | 0.001 | 1.8E-06 | 1.0E-04 | 1.8E-02 | 3.4E-03 | 5.7E-06 | na | - |
| HCH (α) | na | na | 5.0E-03 | - | na | na | 6.0E-03 | - |
| HCH (β) | na | na | 5.0E-03 | - | na | na | 6.0E-03 | - |
| Lindane | na | na | 8.0E-03 | - | na | na | 3.0E-04 | - |
| Heptachlor epoxide | 0.009 | 1.5E-05 | 1.0E-04 | 1.5E-01 | 3.0E-02 | 5.0E-05 | na | - |
| 4,4'-DCBP | 0.009 | 1.4E-05 | na | - | 1.3E-02 | 2.2E-05 | na | - |
| Endrin | 0.001 | 2.0E-06 | 2.0E-04 | 1.0E-02 | 2.8E-03 | 4.7E-06 | 3.0E-04 | 1.6E-02 |
| Pirimicarb | 0.001 | 2.0E-06 | 3.5E-02 | 5.6E-05 | 3.1E-03 | 6.8E-06 | 1.0E-01 | 6.8E-05 |
| Pyrimethanil | 0.003 | 4.2E-06 | 1.7E-01 | 2.5E-05 | 6.3E-03 | 1.1E-05 | 8.5E-01 | 1.2E-05 |
| **HI** |  |  |  | **2.7E-01** |  |  |  |  |

**Table S10.** Hazard quotients (HQ), for toddlers, considering long-term (LT) and short-term (ST) exposure to pollutants, and hazard index (HI) of the OCPs concentrations quantified in fish samples from Hong Kong and Macao markets. EADI: estimated average daily intake; ADI: acceptable daily intake; EMDI: estimated max. daily intake; ArfD: acute reference dose; EC: Environmental concentration; na: not available. (-) Indicates not information available to proceed with the corresponding calculations.

| **Compound** | **HQ-LT (chronic)** | | | | **HQ-ST (acute)** | | | |
| --- | --- | --- | --- | --- | --- | --- | --- | --- |
|  | **Average-EC**  **(mg/kg WW)** | **EADI** | **ADI** | **HQ=EADI/ADI** | **Max-EC**  **(mg/kg WW)** | **EADI** | **ARfD** | **HQ=EADI/ARfD** |
| ∑aldrin+dieldrin | na | na | 1.0E-04 | - | na | na | 3.0E-03 | - |
| Fenobucarb | 2.2E-01 | 7.9E-04 | 6.0E-02 | 1.3E-02 | 4.1E-01 | 1.5E-03 | na | - |
| ∑DDD,DDE,DDT | 6.5E-02 | 2.3E-04 | 5.0E-03 | 4.7E-02 | 1.7E-01 | 6.2E-04 | na | - |
| Endosulfan (α+β+sulphate) | na | na | 6.0E-03 | - | na | na | 2.0E-02 | - |
| Methoxychlor | 9.9E-03 | 3.5E-05 | 1.0E-01 | 3.5E-04 | 1.0E-02 | 3.7E-05 | 5.0E-03 | 7.3E-03 |
| Heptachlor | na | na | 1.0E-04 | - | na | na | na | - |
| HCH (α) | na | na | 5.0E-03 | - | na | na | 6.0E-03 | - |
| HCH (β) | 2.1E-02 | 7.5E-05 | 5.0E-03 | 1.5E-02 | 3.1E-02 | 1.1E-04 | 6.0E-03 | 1.9E-02 |
| Lindane | na | na | 8.0E-03 | - | na | na | 3.0E-04 | - |
| Heptachlor epoxide | 9.7E-02 | 3.4E-04 | 1.0E-04 | **3.4E+00** | 1.1E-01 | 3.9E-04 | na | - |
| 4,4'-DCBP | na | na | na | - | na | na | na | - |
| Endrin | na | na | 2.0E-04 | - | na | na | 3.0E-04 | - |
| Pirimicarb | 3.9E-03 | 1.4E-05 | 3.5E-02 | 3.9E-04 | 4.0E-03 | 1.4E-05 | 1.0E-01 | 1.4E-04 |
| Pyrimethanil | na | na | 1.7E-01 | - | na | na | 8.5E-01 | - |
| **HI** |  |  |  | **3.5E+00** |  |  |  |  |

**Table S11.** Cancer risk (CR), and total cancer risk (TCR) evaluation of insecticide concentrations quantified in fish from Hong Kong and Macao markets. EADI: estimated average daily intake for adults; CSF: cancer slope factor; (na) means compound not detected; (*) means CSF not available; (/) means compound not considered as carcinogenic.

| **Compound** | **EADI** | **CSF** | **CR** |
| --- | --- | --- | --- |
|  | **mg/day/kg BW** | **mg/kg/day** |  |
| ∑aldrin+dieldrin | na | 16 | - |
| Fenobucarb | 4.00E-04 | * | - |
| ∑DDD,DDE,DDT | 1.10E-04 | 0.34 | 3.74E-05 |
| Endosulfan (α+β+sulphate) | na | / | - |
| Methoxychlor | 1.70E-05 | / | - |
| Heptachlor | na | 4.5 | - |
| HCH (α) | na | * | - |
| HCH (β) | 3.50E-05 | 1.8 | 6.30E-05 |
| Lindane | na | 1.3 | - |
| Heptachlor epoxide | 1.60E-04 | 5.5 | 8.80E-04 |
| 4,4'-DCBP | na | * | - |
| Endrin | na | / | - |
| Pirimicarb | 6.50E-06 | * | - |
| Pyrimethanil | na | * | - |
| **TCR** |  |  | 9.80E-04 |

**Table S12** Cancer risk (CR), and total cancer risk (TCR) evaluation of insecticide concentrations quantified in bivalves from Hong Kong and Macao markets. EADI: estimated average daily intake for adults; CSF: cancer slope factor; (na) means compound not detected; (*) means CSF not available; (/) means compound not considered as carcinogenic.

| **Compound** | **EADI** | **CSF** | **CR** |
| --- | --- | --- | --- |
|  | **mg/day/kg BW** | **mg/kg/day** |  |
| ∑aldrin+dieldrin | 7.50E-06 | 16 | 1.20E-04 |
| Fenobucarb | 3.20E-06 | * | - |
| ∑DDD,DDE,DDT | 9.60E-06 | 0.34 | 3.26E-06 |
| Endosulfan (α+β+sulphate) | na | / | - |
| Methoxychlor | 2.10E-06 | / | - |
| Heptachlor | na | 4.5 | - |
| HCH (α) | na | * | - |
| HCH (β) | 4.70E-05 | 1.8 | 8.46E-05 |
| Lindane | na | 1.3 | - |
| Heptachlor epoxide | 9.10E-05 | 5.5 | 5.01E-04 |
| 4,4'-DCBP | 3.30E-05 | * | - |
| Endrin | 1.10E-06 | / | - |
| Pirimicarb | 6.70E-07 | * | - |
| Pyrimethanil | 1.80E-07 | * | - |
| **TCR** |  |  | 7.08E-04 |

**Table S13.** Cancer risk (CR), and total cancer risk (TCR) evaluation of insecticide concentrations quantified in crustaceans from Hong Kong and Macao markets. EADI: estimated average daily intake for adults; CSF: cancer slope factor; (na) means compound not detected; (*) means CSF not available; (/) means compound not considered as carcinogenic.

| **Compound** | **EADI** | **CSF** | **CR** |
| --- | --- | --- | --- |
|  | **mg/day/kg BW** | **mg/kg/day** |  |
| ∑aldrin+dieldrin | 7.40E-06 | 16 | 1.18E-04 |
| Fenobucarb | 6.30E-06 | * | - |
| ∑DDD,DDE,DDT | 6.60E-05 | 0.34 | 2.24E-05 |
| Endosulfan (α+β+sulphate) | 7.60E-06 | / | - |
| Methoxychlor | 1.10E-05 | / | - |
| Heptachlor | 1.80E-06 | 4.5 | 8.10E-06 |
| HCH (α) | na | * | - |
| HCH (β) | na | 1.8 | - |
| Lindane | na | 1.3 | - |
| Heptachlor epoxide | 1.50E-05 | 5.5 | 8.25E-05 |
| 4,4'-DCBP | 1.40E-05 | * | - |
| Endrin | 2.00E-06 | / | - |
| Pirimicarb | 2.00E-06 | * | - |
| Pyrimethanil | 4.20E-06 | * | - |
| **TCR** |  |  | 2.31E-04 |

**Table S14.** Cancer risk (CR), and total cancer risk (TCR) evaluation of insecticide concentrations quantified in fish from Hong Kong and Macao markets. EADI: estimated average daily intake for toddlers; CSF: cancer slope factor; (na) means compound not detected; (*) means CSF not available; (/) means compound not considered as carcinogenic.

| **Compound** | **EADI** | **CSF** | **CR** |
| --- | --- | --- | --- |
|  | **mg/day/kg BW** | **mg/kg/day** |  |
| ∑aldrin+dieldrin | na | 16 | - |
| Fenobucarb | 8.14E-04 | * | - |
| ∑DDD,DDE,DDT | 2.29E-04 | 0.34 | 7.77E-05 |
| Endosulfan (α+β+sulphate) | na | / | - |
| Methoxychlor | 3.25E-05 | / | - |
| Heptachlor | na | 4.5 | - |
| HCH (α) | na | * | - |
| HCH (β) | 8.69E-05 | 1.8 | 1.56E-04 |
| Lindane | na | 1.3 | - |
| Heptachlor epoxide | 3.49E-04 | 5.5 | 1.92E-03 |
| 4,4'-DCBP | na | * | - |
| Endrin | na | / | - |
| Pirimicarb | 1.51E-05 | * | - |
| Pyrimethanil | na | * | - |
| **TCR** |  |  | 2.15E-03 |
